# Multi-scale lodging in rice and wheat: bridging biomechanical traits, phenotypic detection, and intelligent harvesting systems

**DOI:** 10.3389/fpls.2026.1956108

**Published:** 2026-09-28

**Authors:** Tiantian Jing, Xianfei Xia, Zhong Tang, Zhan Su, Zhao Ding

**Affiliations:** 1 School of Agricultural Engineering, Jiangsu University, Zhenjiang, China; 2 Nanjing Institute of Agricultural Mechanization, Ministry of Agriculture and Rural Affairs, Nanjing, China; 3 Key Laboratory of Crop Harvesting Equipment Technology of Zhejiang Province, Jinhua University of Vocational Technology, Jinhua, China

**Keywords:** biomechanics, crop phenotyping, deep learning, intelligent harvesting, lodging resistance, remote sensing

## Abstract

Lodging severely compromises the productivity and harvestability of rice and wheat, yet the mechanistic links among molecular regulation, tissue structure, whole-plant mechanical failure, field phenotypes, and harvesting responses remain insufficiently integrated. This review establishes a mechanically consistent, plant-centered, cross-scale framework that connects genetic and biochemical regulation to quantifiable engineering parameters, including bending moment, section modulus, flexural rigidity, and resisting moment, and links these parameters to canopy-scale structural and optical phenotypes and ultimately to harvestability. We show that tissue-level material properties and organ-scale geometry jointly determine root anchorage, flexural rigidity, breaking resistance, and whole-plant lodging behavior. We further explain how mechanical failure reorganizes canopy geometry and optical properties, generating structural and spectral phenotypes that can be quantified through field phenotyping, unmanned aerial vehicle (UAV) remote sensing, three-dimensional sensing, and machine- and deep-learning approaches. Finally, we identify root–shoot mechanical coupling, standardized biomechanical measurements, cross-environment model validation, and the integration of perception with adaptive harvesting control as major priorities for future research. By unifying plant biology, mechanics, digital phenotyping, and engineering action within a common physical framework, this review provides a basis for lodging-resistant breeding, field monitoring, and intelligent crop management.

## Graphical abstract

**Figure:**
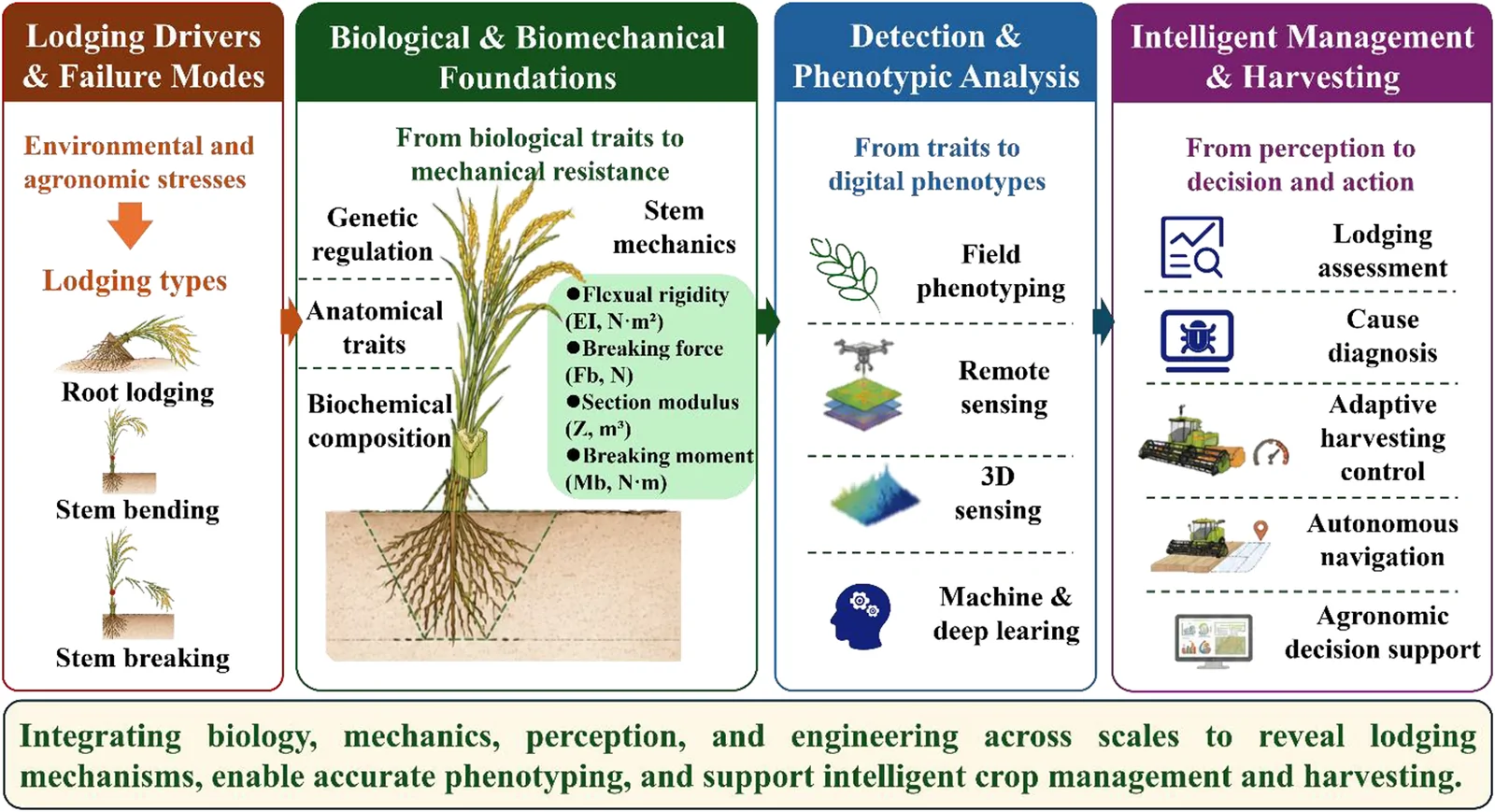

## 1 Introduction

Rice and wheat are two pillars of global food security and provide staple foods for more than half of the world’s population (Fernandez-Jibaja et al., 2025; Mullangie et al., 2024; Yang et al., 2025). With the global population projected to reach 9.7 billion by 2050 while arable land continues to decline, increasing cereal yield per unit area has become imperative (Quan et al., 2025). Among the many factors constraining yield improvement, lodging remains one of the most persistent and destructive physical limitations (Gao et al., 2026; Liao et al., 2023; Sun et al., 2025).

Lodging frequently occurs during grain filling in rice and wheat, although its timing and incidence vary with crop species, cultivar, management practices, and prevailing weather conditions (Gao, 2018; Yan et al., 2017). Modern intensive cultivation practices can increase yield but may also weaken stem mechanical strength, thereby increasing lodging risk (Mullangie et al., 2024; Yang et al., 2025). Global environmental change further amplifies this threat: elevated tropospheric ozone concentrations and climate warming have been shown to impair stem mechanical properties and increase susceptibility to structural failure (He et al., 2020; Hu et al., 2023; Shao et al., 2024; Zhang et al., 2023b; Zhou et al., 2025). Although agronomic innovations such as drone-ordered hill direct seeding can improve population-level lodging resistance (Shi et al., 2021; Wang et al., 2026b), lodging remains a serious constraint.

The effects of lodging on cereal production are multidimensional (**Figure 1**), encompassing yield loss, quality deterioration, harvesting difficulty, and economic loss. At the yield level, lodging causes canopy closure and damages internal vascular bundles (Ren et al., 2021; Wang et al., 2024b), disrupting long-distance assimilate transport. In extreme cases, rice yield losses can reach 80% (Kumar et al., 2024; Lv et al., 2022; Zhang et al., 2025), while wheat yield losses can reach 61% (Li et al., 2026a). At the quality level, lodging disrupts grain filling, substantially reducing the milling and eating quality of rice and impairing the dough rheological properties of wheat (Gao et al., 2026). At the economic level, intertwined and entangled stems reduce mechanical harvesting efficiency and increase threshing losses, creating a substantial economic burden for fully mechanized rice and wheat production (Li et al., 2026a; Ma et al., 2025; Tang et al., 2020).

**Figure 1:**
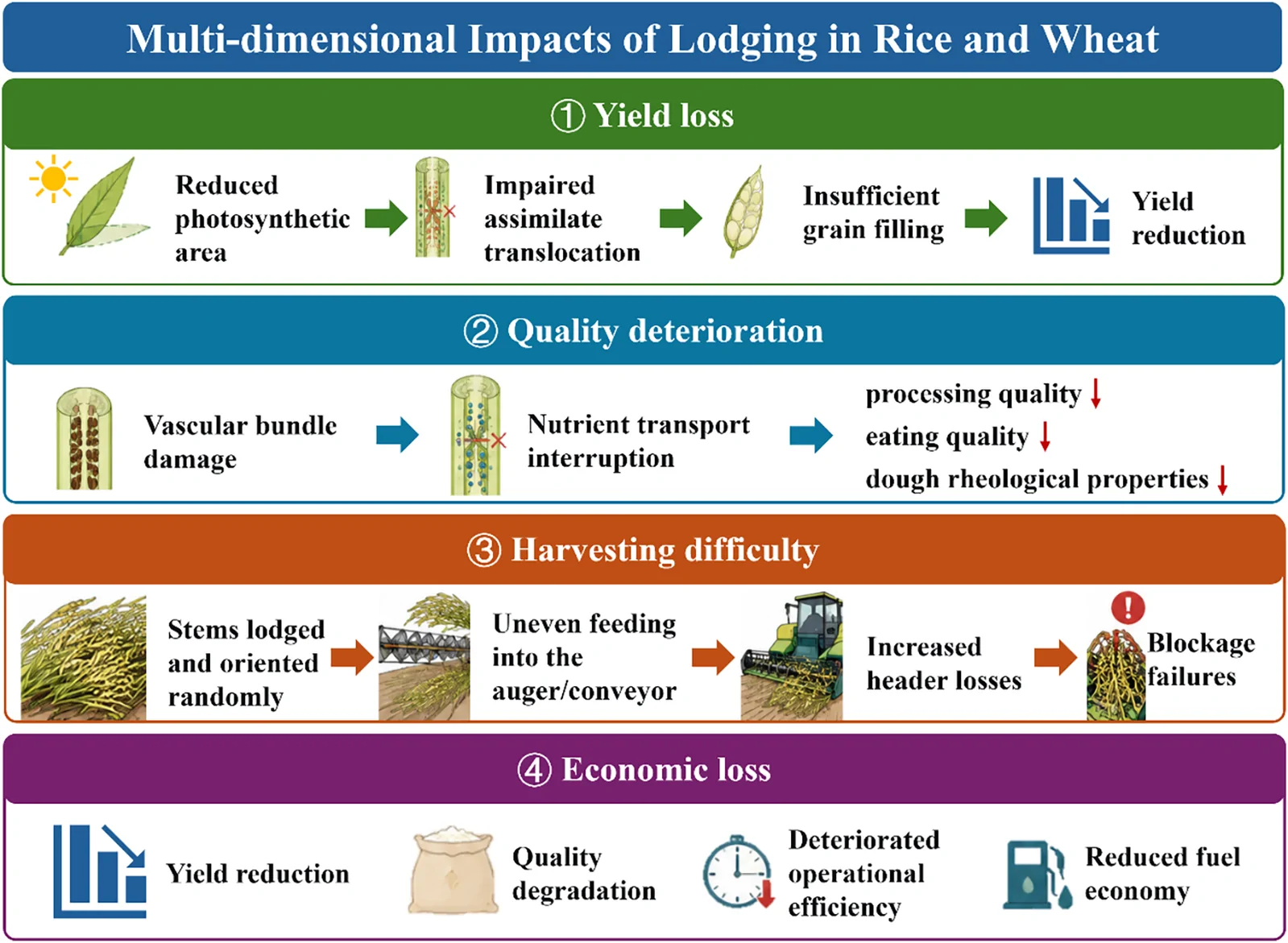
Multidimensional impacts of lodging.

Lodging also directly disrupts harvesting operations and represents a critical engineering bottleneck to full mechanization (Zhuang and Li, 2023). Under upright crop conditions, modern combine harvesters can generally maintain relatively low total loss rates (Lian et al., 2021; Liang et al., 2016; Tang et al., 2019). Lodging, however, fundamentally changes the spatial and mechanical interaction between the harvester and the crop (Tang et al., 2021). Low-lying stems with disordered orientations increase cutting losses, reduce the reel’s lifting and guiding effectiveness, and cause uneven feeding into the auger, frequently resulting in severe blockage (Hao et al., 2022). Operators must therefore often reduce forward speed and manually adjust working components, increasing harvesting time while reducing operational efficiency and fuel economy (Liang et al., 2017).

Harvesting losses increase with lodging severity, and the angle between the main stem and the ground is a key parameter governing harvesting difficulty. When the inclination angle exceeds 60°, the increase in losses is limited; at angles of 30°–60°, the header must operate close to the ground and total losses rise to 5%–10% (Xie et al., 2023); below 30°, many stems fall beneath the operational limit of the cutting blade, and total losses can exceed 30% in extreme cases (Yao et al., 2025; Zhang et al., 2026b). Moreover, microtopography and variable wind directions often produce a spatial mosaic of lodging severities within the same field (Wu and Wei, 2019; Zhu et al., 2025). This heterogeneity makes it difficult for the header and reel to respond effectively using a single parameter set and substantially complicates loss control (Liu et al., 2025a; Zhou et al., 2026b).

A major source of lodging-related harvesting losses is the lack of prior information on the spatial distribution and severity of lodging. In conventional harvesting operations, parameter adjustment depends heavily on the operator’s visual judgment and experience (Zhang et al., 2024b). As a result, the harvester operates reactively: corrective action is typically taken only after cutting losses or blockages have occurred, by which time substantial losses may already have accumulated. This limitation reflects the absence of a front-end sensing stage in the harvesting information chain, which deprives downstream decision-making of feedforward information (Feng et al., 2024a; Guo et al., 2025). Addressing this gap requires three coordinated capabilities, namely real-time lodging perception, adaptive control, and autonomous navigation, which correspond to the perception, control, and navigation layers of the information chain and together enable the transition from experience-based operation to sensor-driven intelligent harvesting (Chen et al., 2025b; Pan et al., 2025).

In summary, the constraints imposed by lodging on rice and wheat production span three tightly interconnected domains: biological mechanisms, phenotyping and sensing, and mechanized harvesting. These domains form a causal cross-scale hierarchy rather than independent research themes. Genetic and biochemical regulation shapes tissue anatomy and material properties; these tissue-level traits, together with root and stem geometry, determine organ-scale mechanical resistance; mechanical resistance governs whole-plant lodging failure; and the resulting reorganization of canopy structure and optical properties generates field-scale structural and spectral phenotypes that can be detected and translated into management and harvesting decisions. Accordingly, this review follows this hierarchy, progressing from lodging mechanisms and associated traits through genetic and biomechanical determinants to remote-sensing phenotypes, machine- and deep-learning-based recognition, and finally real-time sensing, adaptive control, and autonomous navigation. By explicitly linking mechanism, phenotype, perception, and action, the review clarifies the connections among plant biology, mechanics, remote sensing, computer vision, and agricultural equipment engineering and provides a cross-disciplinary basis for lodging-resistant breeding, damage assessment, and intelligent harvesting. While each individual link in this chain is supported by previous studies to varying degrees, the explicit integration of these levels into a single mechanical framework represents the conceptual contribution of this review; where the supporting evidence remains limited or emerging, this is stated explicitly and the relevant discussion is framed as a future perspective.

## 2 Scope and literature selection

Relevant literature was identified through searches of Web of Science, Scopus, ScienceDirect, and Google Scholar, covering publications available through 2026. Recent advances were emphasized while earlier landmark studies essential to the development of lodging biology and biomechanics were retained. Representative search terms included combinations of “rice lodging,” “wheat lodging,” “lodging resistance,” “root lodging,” “stem lodging,” “biomechanics,” “breaking moment,” “phenotyping,” “UAV remote sensing,” “deep learning,” “lodging detection,” “adaptive harvesting,” and “autonomous navigation.” Priority was given to peer-reviewed studies directly relevant to the scope of this review, particularly those providing mechanistic, quantitative, methodological, or engineering evidence. The selected literature covers biological mechanisms, biomechanical characterization, phenotyping and sensing, intelligent recognition, and harvesting applications; publications lacking direct relevance to lodging or sufficient methodological detail were used only as background references. This review focuses on rice and wheat. Evidence from other cereals is included only when it establishes a mechanism or method not yet demonstrated in rice or wheat, with the species identified at the point of use.

## 3 Lodging mechanisms and associated traits

This section first distinguishes lodging by the location and mode of mechanical failure and then examines its determinants across hierarchical levels. Root lodging is treated as failure of the root–soil anchorage system, whereas stem lodging is treated as bending or breaking of the basal internodes. Within stem lodging, organ-scale geometry and loading are distinguished from tissue-level anatomical and biochemical determinants of material strength. This organization separates failure mode, mechanical traits, and biological determinants while preserving their causal relationships, thereby providing a mechanistic foundation for the subsequent discussion of genetic regulation and biomechanical performance.

### 3.1 Root lodging and associated traits

Based on the location of mechanical failure, lodging can be classified into root lodging, stem bending, and stem breaking. Mechanically, root lodging occurs when the anchorage resisting moment of the root–soil complex (*M*_anchor_, N·m) is lower than the wind-induced overturning moment acting on the aboveground plant (*M*_wind_, N·m), resulting in irreversible rotation of the plant around the root–shoot junction (Mullangie et al., 2024). A key intrinsic determinant of root anchorage strength is root system architecture, particularly the spread diameter of the root plate. Biomechanical models indicate that the failure moment of crop anchorage increases strongly with root plate spread diameter, making greater root-plate spread an important determinant of resistance to wind-induced overturning (Piñera-Chavez et al., 2016). In direct-seeded rice cultivation, root lodging is particularly common because of restricted development of shallow root systems; however, empirical evidence demonstrates that agronomic interventions such as mechanical furrowing can significantly increase overall root biomass and the proportion of structural deep roots, thereby enhancing the anchorage performance of the system (Zhang et al., 2021).

Root anchorage force depends not only on intrinsic morphology but is also strongly influenced by external physical conditions such as rhizosphere soil moisture and compaction status. Here, anchorage force (*F*_anchor_, N) refers to a directly measured force-based trait and should be distinguished from the anchorage resisting moment (*M*_anchor_, N·m). When a force-based test is used to characterize rotational resistance, the corresponding resisting moment should be calculated from the applied force and its effective lever arm, i.e., $M_{\text{anchor}}=F_{\text{anchor}}l_{\text{anchor}}$. When the surface soil is at or near saturation, the sharp loss of matric suction markedly reduces soil shear strength, creating a particularly vulnerable period for root lodging (Piñera-Chavez et al., 2016). Evidence from winter barley indicates that moderate soil compaction may enhance anchorage by increasing soil resistance and strengthening root–soil interaction, whereas excessive compaction can restrict root elongation and reduce root-system development. Therefore, soil mechanical conditions should be interpreted in terms of their net effects on both root growth and anchorage rather than penetration resistance alone (Scott et al., 2005; Lima et al., 2025). Although this evidence was obtained in winter barley, it illustrates a soil–root interaction principle that is transferable to rice and wheat rather than a species-specific result.

At the whole-plant level, root lodging resistance reflects the mechanical interaction between the belowground anchorage system and the aboveground stem–canopy morphology. Multivariate analysis has also identified center-of-gravity height (CGH) as an important aboveground determinant of lodging susceptibility. Ren et al. (2026a) termed the ratio of single-stem elasticity (SSE) to CGH an integrative lodging index (LI_3_): $LI_{3}=SSE/CGH$. In their measurement system, SSE was reported in kPa and CGH in cm, giving LI_3_ a dimensional form of kPa·cm^−1^. Strictly speaking, LI_3_ is therefore not a dimensionless lodging index but an empirical metric with physical units; its numerical value depends on the units selected, which limits direct comparison among studies. Within the measurement system used by Ren et al. (2026a), a higher LI_3_ value indicates greater lodging resistance.

Because root anchorage depends on the three-dimensional configuration of the root system and its interaction with the surrounding soil, mechanistic analysis requires quantitative characterization of root architecture while preserving root–soil geometry as far as possible. This creates a methodological gap between destructive root excavation and automated quantitative analysis. Non-destructive three-dimensional (3D) imaging provides an important intermediate solution. Magnetic resonance imaging (MRI) enables non-invasive 3D visualization and quantitative characterization of root system architecture in soil, including root length, diameter, spatial distribution, and growth angles (van Dusschoten et al., 2016). Complementary to MRI, X-ray microcomputed tomography (μCT), coupled with automated root-segmentation approaches such as RooTrak, enables reconstruction of 3D root architecture directly from soil–root image volumes (Mairhofer et al., 2012). These techniques are particularly relevant to lodging because root spread, rooting depth, and 3D root distribution influence the anchorage behavior of the root–soil complex. Quantitative traits extracted from MRI and μCT therefore provide a structural basis for parameterizing biomechanical models of root anchorage and overturning failure.

Imaging alone, however, does not complete the transition from root structure to mechanically useful phenotypes; high-throughput lodging research also requires efficient extraction of quantitative traits from complex image data. Deep learning-based computer vision provides the next analytical step by enabling automated root segmentation and trait extraction. For example, the Dual-Guided Asymmetric MP-Former, developed specifically for fine and overlapping rice root systems, substantially reduced errors in morphological extraction and achieved precise pixel-level recognition under complex backgrounds (Liang et al., 2026a). In future work, integrating non-destructive imaging with automated trait extraction will be important not only for disentangling the confounding effects of root and stem lodging (Mullangie et al., 2024), but also for translating root structural information into parameters for mechanical models and advancing cross-species dynamic lodging prediction toward field application (Lima et al., 2025).

### 3.2 Stem lodging and associated traits

Stem lodging is the most prevalent and destructive form of lodging in cereal production and refers to the permanent loss of upright posture caused by basal stem bending or breaking under wind and rain loading (Li et al., 2026a). Based on mechanical failure mode, stem lodging can be divided into two subtypes. Bending-type lodging is characterized by plastic deformation of the basal internodes while the tissue remains intact and is associated primarily with morphological traits such as excessive plant height and an elevated center of gravity. Stem-breaking lodging involves structural fracture of the first to third basal internodes and directly reflects insufficient tissue mechanical strength (Li et al., 2026a). **Figure 2** illustrates the mechanical loading states and failure modes of root and stem lodging in rice and wheat.

**Figure 2:**
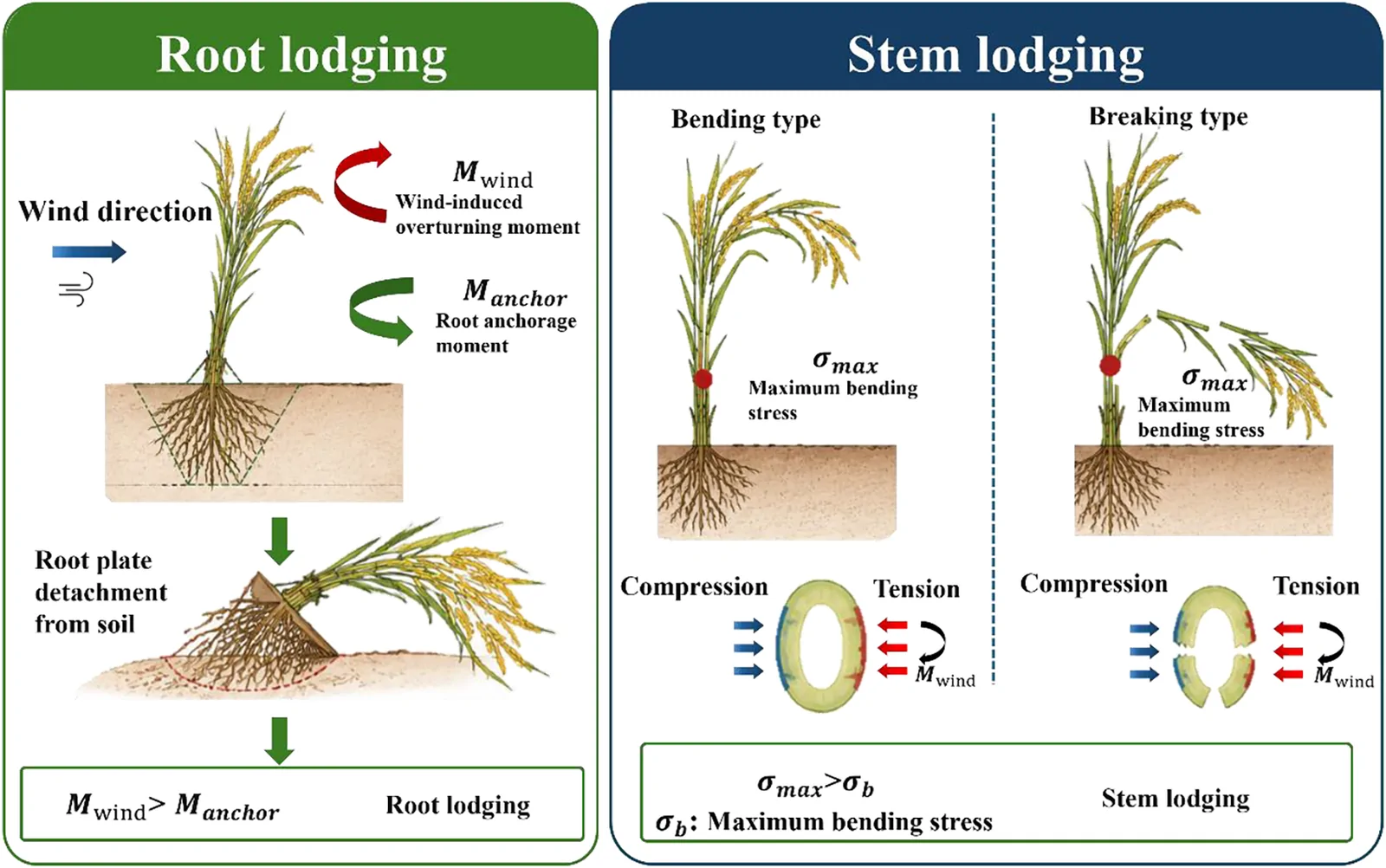
Mechanical loading and failure modes of root lodging and stem lodging in rice and wheat. *M*_wind_ denotes the wind-induced overturning or bending moment (N·m), and *M*_anchor_ denotes the resisting moment provided by the root–soil anchorage system (N·m). Root lodging is expected when *M*_wind_ exceeds *M*_anchor_. For stem lodging, σ_max_ denotes the maximum bending stress generated in the basal internode (Pa or MPa), whereas σ*_b_* denotes the corresponding failure stress of the stem tissue (Pa or MPa). Stem structural failure occurs when σ_max_ approaches or exceeds σ*_b_*.

As illustrated in **Figure 2**, root and stem lodging differ in both the location of mechanical failure and the structure providing resistance. Root lodging occurs when the wind-induced overturning moment (*M*_wind_, N·m) exceeds the resisting anchorage moment of the root–soil complex (*M*_anchor_, N·m), whereas stem failure occurs when the maximum bending stress in a basal internode (σ_max_, Pa or MPa) reaches or exceeds its failure stress (σ*_b_*, Pa or MPa). This distinction implies different mechanical targets for improving lodging resistance: enhancing root–soil anchorage for root lodging versus increasing stem geometric resistance and tissue strength for stem lodging.

The main morphological determinants of stem lodging resistance include center-of-gravity height, basal internode dimensions, and section modulus. Mechanically, the basal bending moment (M, N·m) is determined by the external force (F, N) and its effective lever arm (l, m), such that M=Fl. An increase in center-of-gravity height therefore lengthens the effective lever arm of aboveground loading and can increase the bending moment acting on the basal internodes. Basal internode development is also highly sensitive to the canopy microenvironment: canopy closure and reduced light transmission in the lower canopy can promote excessive internode elongation in both rice and wheat, thereby increasing the effective lever arm and reducing lodging resistance. Conversely, larger internode outer diameter and culm wall thickness alter cross-sectional geometry, increasing the second moment of area ((I), m^4^) and section modulus ((Z=I/c), m^3^). A larger (Z) reduces bending stress at a given bending moment, whereas a larger (I), together with tissue Young’s modulus ((E), Pa), increases flexural rigidity ((EI), N·m^2^) (Zhang et al., 2025). These morphological traits are genetically regulated but also show substantial phenotypic plasticity in response to management practices such as water and fertilizer regimes (Feng et al., 2024b).

Recent multi-trait field phenotyping further supports evaluating lodging resistance through coordinated structural and mechanical traits rather than through a single descriptor. Across 79 japonica accessions evaluated over two years under dry direct seeding, Tang et al. (2026) showed that strong lodging resistance was associated with coordinated reductions in plant height and center-of-gravity height together with greater basal-internode bending stress; structural equation modeling further identified culm dry weight and inner diameter as major contributors to lodging variation. These results illustrate how integrated phenotyping can link whole-plant loading with organ-scale mechanical resistance and help define lodging-resistant breeding ideotypes.

These organ-scale morphological and geometric traits govern how external loads are transmitted to the basal internodes, but geometry alone does not determine the stem failure threshold. Resistance to bending and breaking also depends on tissue-level material properties arising from anatomical organization and biochemical composition. Accordingly, Section 3.3 considers these tissue-level determinants separately from the organ-scale mechanics discussed here.

These organ-scale loading and resistance traits have also been incorporated into empirical lodging indices in the agronomic literature. Some published indices combine a bending-moment-related load term with a force-based measure of stem breaking resistance (Niu et al., 2012). Such indices can be useful empirical descriptors, but they should not be interpreted as dimensionless mechanical ratios unless the measured breaking force is converted to a resisting moment using the corresponding test lever arm. Modern high-yield cultivation systems also involve an inherent trade-off between single-stem mechanical strength and population density: dense planting intensifies competition for light, producing thinner stems with lower mechanical strength (Mizuta et al., 2023). A systematic evaluation of stem lodging resistance must therefore consider both the material properties of individual stems and the spatial configuration of the canopy, seeking a mechanically informed balance between individual load-bearing capacity and population yield potential.

### 3.3 Anatomical and biochemical determinants of stem strength

At the tissue level, stem strength is jointly determined by anatomical structure and biochemical composition: anatomy provides the spatial framework for mechanical load bearing, whereas biochemical components confer material stiffness and toughness (Ren et al., 2026a). These two sets of traits remain dynamically coupled from internode development through grain filling.

At the anatomical level, the vascular bundle system and sclerenchyma tissue form the primary structural basis of resistance to bending. Mechanical testing and finite element simulations reveal a distinct spatial gradient in the stiffness of large vascular bundles in rice stems, with stiffness decreasing from the basal to the upper regions and from the outer to the inner tissues. Vascular bundle geometry and spatial distribution contribute to the second moment of area (*I*), which, together with tissue elastic properties, determines the overall flexural rigidity (*EI*) of the stem (Shi et al., 2025). In addition, the number of sclerenchyma cell layers distributed on the inner side of the epidermis and their cell wall thickness are major histological determinants of breaking resistance; experimental evidence shows that uniconazole treatment can increase sclerenchyma tissue thickness by approximately 18% to 30% (Lv et al., 2022; Zhu et al., 2023).

Although conventional histological microscopy provides high-resolution information on vascular bundle organization, sclerenchyma development, and cell-wall thickness, its low throughput limits large-scale genetic analysis. Emerging high-throughput anatomical phenotyping platforms, such as AnatomyArray, combine multiplexed tissue sectioning with deep learning-based image analysis to automate the quantification of cellular and tissue-level anatomical traits at scale (Cheng et al., 2025). Such approaches could substantially accelerate associations between stem anatomical traits, genetic loci, and mechanical parameters, thereby linking microscopic tissue architecture with population-scale genetic analysis and lodging-resistance breeding.

Recent micro-phenotyping studies further extend this capacity to lodging-relevant stem structures. SEM-based digital phenotyping quantified 18 straw cross-sectional traits across 147 rice accessions and linked cell-wall and internode features to genetic variation associated with stem architecture and lodging resistance (Mohamadiaza et al., 2026). More recently, a biologically informed deep-learning framework automated the segmentation of rice stem vascular bundles and the extraction of 14 stem traits from 686 cross-sections, greatly increasing phenotyping throughput while retaining strong concordance between image-derived traits and genetic association signals (Li et al., 2026d). Although these approaches do not directly score field lodging, they provide scalable microstructural phenotypes that can serve as mechanistic inputs for evaluating lodging resistance.

Secondary-cell-wall polymers form the molecular basis of stem material strength. Among them, lignin content and monomer composition, specifically p-hydroxyphenyl (H), guaiacyl (G), and syringyl (S) units, contribute substantially to the mechanical properties of stem tissues. Variation in the S/G ratio has been associated with differences in stem mechanical performance; however, transcriptomic and metabolomic analyses in hulless barley indicate that the direction and magnitude of this relationship depend on tissue context, total lignin content, lignin–polysaccharide cross-linking, and the broader cell-wall architecture (Wang et al., 2025a; Yu et al., 2021). Lignin and cellulose accumulation also shows strong environmental plasticity: low-light stress suppresses key enzymes in the phenylpropanoid pathway and markedly reduces stem breaking strength (Qin et al., 2025), whereas delayed sowing or supplemental red light promotes structural carbohydrate accumulation, alters lignin monomer composition, and improves cellulose crystallinity (Li et al., 2025a; Wang et al., 2025a). Within the cell wall, cellulose microfibrils provide the tensile framework, while hemicelluloses such as xylan act as bridging molecules; disruption of their synthesis pathways, as in the Fragile culm19 (FC19) mutation, causes broad mechanical remodeling of cell-wall polysaccharides (Dang et al., 2023).

In addition to organic polysaccharides, mineral elements such as silicon and potassium can further reinforce stem rigidity through deposition in the cell wall. Under optimized nitrogen management, silicon accumulates in the epidermis and vascular bundle sheaths (Zhou et al., 2024) and can co-deposit at the nanoscale with hemicellulose and lignin to form a strong “silica–polysaccharide–lignin” ternary composite network (Miao et al., 2023).

Stem mechanical properties also vary dynamically over development because of an intrinsic physiological trade-off in carbon allocation. During late grain filling, substantial amounts of non-structural carbohydrates are transported to the panicle; if this remobilization outpaces the net accumulation of structural compounds in the stem, a period of heightened lodging susceptibility can develop (Li et al., 2025a; Lv et al., 2022). From a systems-biology perspective, stem lodging resistance is therefore not a simple sum of static traits but a dynamic outcome of the spatiotemporal coupling among anatomical differentiation, polysaccharide assembly, and elemental deposition (Ren et al., 2026a; Zhu et al., 2023).

## 4 Lodging resistance: from genetic architecture to mechanical performance

Having established the mechanical failure modes and their organ- and tissue-level determinants, the next question is how these traits are biologically regulated and translated into mechanical performance. Section 4 therefore connects upstream genetic regulation with downstream mechanical behavior. Section 4.1 examines genes and signaling pathways that modify plant architecture and cell-wall-related traits, whereas Section 4.2 formalizes how these biologically regulated changes are expressed as measurable mechanical resistance and failure criteria.

### 4.1 Genetic basis of lodging resistance

Genetic improvement of lodging resistance has been a major achievement of twentieth-century cereal breeding, largely through the discovery and deployment of semi-dwarfing genes such as *sd1* in rice and *Rht* in wheat. These “Green Revolution” genes reduce plant height and reshape crop mechanical architecture by altering gibberellin (GA) synthesis or signal transduction (Li et al., 2026a; Niu et al., 2022). Recent mechanistic studies have shown that the transcription factor *OsTCP4* negatively regulates *SD1* transcription (Ruan et al., 2026), while *Rht* alleles can produce tissue-specific truncated proteins through translational re-initiation (Van De Velde et al., 2021). However, semi-dwarfing genes can involve physiological trade-offs: *sd1* induces pleiotropic changes in tillering and biomass allocation in addition to reducing plant height, suggesting that excessive reliance on whole-plant dwarfing may compromise yield potential (Jia et al., 2020).

**Figure 3** provides a representative example of how spatial gene expression relates to the regulation of plant architecture. *OsTCP4* is preferentially expressed in stem nodes and tiller bases, and the OsTCP4 protein is localized in the nucleus, consistent with its role as a transcriptional regulator of *SD1* and plant height. This spatial expression pattern highlights the importance of considering the tissue context of growth-regulatory genes rather than evaluating lodging-resistance strategies solely in terms of whole-plant height reduction.

**Figure 3:**
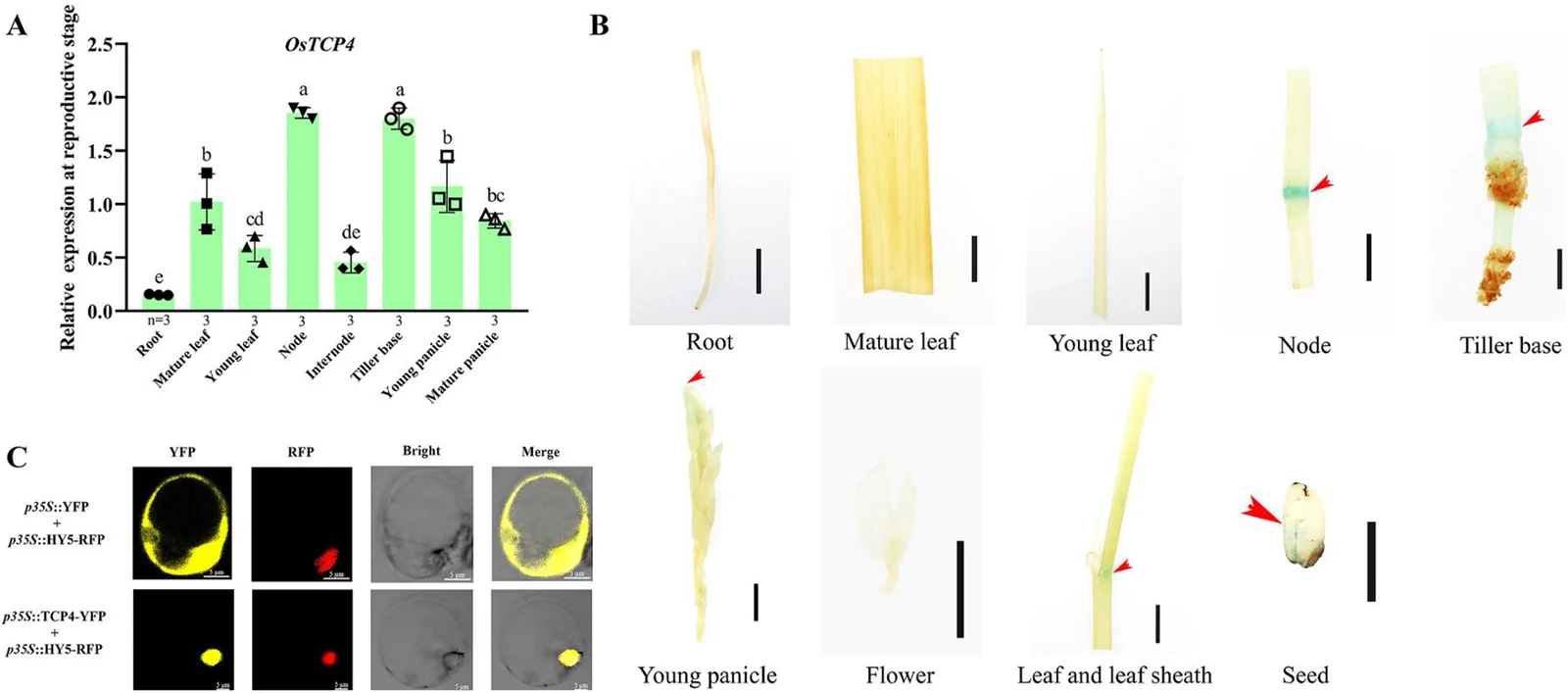
Tissue-specific expression and subcellular localization of OsTCP4 in rice, illustrating the spatial basis of its role in plant-height regulation. **(A)** Relative expression levels of *OsTCP4* in different tissues of wild-type Nipponbare plants determined by RT-qPCR. *OsActin1* was used as the internal reference gene. Values are means ± SD (*n* = 3); different letters indicate significant differences among tissues (*P* < 0.05). **(B)** GUS staining of *Pro-OsTCP4::GUS* transgenic rice showing preferential promoter activity in the node and tiller base. Scale bars = 5 mm. **(C)** Subcellular localization of OsTCP4 in rice protoplasts. OsTCP4-YFP fluorescence colocalizes with the nuclear marker HY5-RFP, indicating nuclear localization of OsTCP4, whereas the YFP control is broadly distributed within the cell. Together, the preferential expression of *OsTCP4* in stem-associated tissues and its nuclear localization support its function as a transcriptional regulator of plant height through modulation of the *SD1* pathway. Reproduced from Ruan et al. (2026).

To overcome the limitations of a single dwarfing strategy, current research increasingly targets genes that directly enhance stem strength or produce localized dwarfing. For example, the rice *SBI* gene confers “basal internode-specific dwarfing” through localized degradation of bioactive GA, thereby reducing the pleiotropic penalties associated with whole-plant dwarfing (Liu et al., 2018). Similarly, cloning of the glycosyltransferase gene *DBC3* has demonstrated that maintenance of cell-wall polysaccharide synthesis makes an independent genetic contribution to stem mechanical strength (Dai et al., 2026). In wheat, natural variation at an ethylene-responsive factor locus such as *TaERF-A1* confers a lodging-resistant plant architecture by fine-tuning hormone signaling without substantially reducing plant height (Li et al., 2025b).

At the quantitative-trait and population-genetic levels, genome-wide analyses provide a broader view of the genetic architecture of lodging resistance. Long-term breeding has produced measurable gains: over the past 50 years, dominant wheat cultivars in China’s Yellow-Huai River Valleys Winter Wheat Zone have shown significant reductions in plant height and center of gravity, together with an approximately 19% increase in basal breaking strength (Zhang et al., 2020b). However, quantitative trait locus (QTL) analyses also indicate that chromosomal regions containing certain dwarfing genes, such as *Rht-5, may be associated with* linkage drag affecting grain number per spike (Daoura et al., 2014). Artificial lodging experiments in foxtail millet have further quantified the yield penalty associated with lodging and shown that lodging during grain filling is particularly damaging to yield components (Tian et al., 2018). Although these experiments were conducted in foxtail millet, the finding that lodging during grain filling causes the greatest yield penalty provides a transferable reference for rice and wheat. Modern breeding for lodging resistance therefore increasingly emphasizes coordinated optimization of genetic traits and agronomic management; for example, combining modern semi-dwarf hybrid rice with alternate wetting and drying (AWD) irrigation can couple high yield potential with strong lodging resistance (Gao et al., 2024; Gu et al., 2025).

In summary, lodging resistance in rice and wheat is shaped by the GA pathway (Ruan et al., 2026; Van De Velde et al., 2021), cell-wall modification (Dai et al., 2026), and crosstalk among multiple hormone pathways (Li et al., 2025b; Liu et al., 2018). Future genetic improvement should move beyond single-gene introgression by combining validated genome editing of lodging-related genes with genomic prediction of mechanically relevant traits, enabling coordinated optimization of plant architecture, stem strength, and yield potential (Niu et al., 2022; Li et al., 2026a; Zhao et al., 2025b). These genetic and biochemical changes influence lodging resistance through measurable structural and mechanical phenotypes. Accordingly, the following section translates biologically regulated variation into physical parameters such as stem geometry, section modulus, flexural rigidity, bending stress, and resisting moment, all of which determine macroscopic biomechanical stability.

### 4.2 Mechanical principles of stem strength

Stem lodging can be viewed as the mechanical failure of a load-bearing structure. From an engineering-mechanics perspective, an upright cereal stem can be approximated as a cantilever beam fixed near its base (Kashiwagi et al., 2010). An external force (*F*, N) acting at an effective lever arm (*l*, m) generates a bending moment *M* = *Fl* (N·m). Accordingly, a wind-induced force *F*_wind_ produces a basal bending moment $M_{\text{wind}}=F_{\text{wind}}l_{\text{wind}}$. In contrast, a mechanical bending test may directly report a breaking force (*F_b_*, N); to compare this measurement with the applied bending moment, it should be converted to a breaking or resisting moment, $M_{b}=F_{b}l_{b}\left(\text{N}\cdot \text{m}\right)$, where *l_b_* is the distance between the force application point and the failure section. A dimensionally consistent moment-based failure criterion can therefore be expressed as $M_{\text{wind}}\geq M_{b}$. At the cross-sectional level, the corresponding maximum bending stress is $\sigma_{\text{max}}=M_{\text{wind}}/Z\left(\text{Pa}\right)$, where Z=I/c is the section modulus (m^3^), *I* is the second moment of area (m^4^), and *c* is the distance from the neutral axis to the outermost fiber (m). In contrast, flexural rigidity *EI* (N·m^2^) characterizes resistance to elastic bending deformation and is the product of Young’s modulus *E* (Pa) and *I*. Thus, breaking force, bending moment, bending stress, section modulus, and flexural rigidity represent distinct but mechanically related quantities and should not be used interchangeably.

Various empirical lodging indices and safety factors have been proposed in agronomic studies (Ding et al., 2021; Luo et al., 2023). In the dimensionally consistent mechanical framework adopted here, applied and resisting quantities are expressed at the same physical level. A moment-based lodging index can therefore be written as $LI<M_{\text{wind}}/M_{b}$, whereas the corresponding lodging safety factor is $SF=M_{b}/M_{\text{wind}}$. Both are dimensionless. Accordingly, *LI* < *I* (or equivalently *SF* > 1) indicates that the resisting moment exceeds the applied bending moment, whereas *LI* ≥ 1 indicates that the applied moment has reached or exceeded the mechanical resistance. Empirical studies show that optimized planting patterns can improve the light environment at the canopy base, promoting the accumulation of cellulose and other substances in basal internodes, and thereby increasing the lodging safety factor reported for wheat by up to 26.5% (Luo et al., 2023). Furthermore, high-throughput QTL mapping analyses have confirmed that breaking force and section modulus are major mechanical contributors to variation in lodging-resistance phenotypes (Mamidi et al., 2026).

Stem mechanical properties are not static constants but dynamic phenotypes regulated by factors operating across scales. At the molecular and pathological levels, disruption of cell-wall polysaccharide synthesis pathways, such as *DBC3* and *FC19*, or enzymatic degradation of basal cell walls by sheath blight pathogens directly weakens tissue material strength (Dai et al., 2026; Dang et al., 2023; Wu et al., 2012). At the environmental and management levels, elevated ozone exposure and excessive nitrogen fertilization reduce stem mechanical stability (Shao et al., 2024; Zhang et al., 2016), whereas optimization of canopy spatial distribution through drone-ordered hill direct seeding or heterogeneous population configurations can partly offset these losses (Hu et al., 2024; Wang et al., 2026b). Stem-level mechanical properties therefore integrate the long-term effects of internal biochemical composition and external environmental perturbations (Li et al., 2026b).

Within this review, mechanical principles provide the central bridge between biological regulation and observable phenotypes. They translate molecular genetic and biochemical traits into measurable mechanical properties (Lv et al., 2022) and provide reference quantities for calibrating and validating remote-sensing estimates and adaptive tactile sensing (Chen et al., 2025b). Current research is increasingly moving toward multiscale finite element simulation and the use of mechanical parameters in intelligent breeding decisions. However, cross-study comparability remains limited by substantial heterogeneity in field-testing protocols, including differences in growth stage, loading mode, and moisture status. Establishing standardized protocols for stem biomechanical testing is therefore essential for linking theoretical mechanics with high-throughput intelligent phenotyping. The next scale transition is from intrinsic mechanical resistance to the observable consequences of mechanical failure at the canopy and field levels. Rather than inferring intrinsic mechanical properties directly from remote-sensing signals, the key task is to establish calibrated relationships between mechanically defined failure states and the structural and spectral phenotypes produced by lodging.

## 5 UAV remote sensing and spectral identification of lodging

Once mechanical failure occurs, an initially upright stem–canopy system bends or collapses, reorganizing canopy geometry and leaf orientation and thereby altering the optical scattering regime. Reduced multiple scattering within the disrupted canopy contributes to lower near-infrared (NIR) reflectance and decreases in NIR-sensitive vegetation indices such as NDVI, whereas increased exposure of stems, senescent leaves, and background surfaces modifies visible-band reflectance. Structural products derived from photogrammetry or light detection and ranging (LiDAR) provide a complementary geometric description: reductions in canopy height and reconstructed stem inclination serve as spatially explicit proxies for lodging-induced displacement and posture change. These remotely sensed traits should therefore be interpreted not as direct measurements of intrinsic mechanical properties such as flexural rigidity (EI, N·m^2^), breaking force (Fb, N), breaking/resisting moment (Mb, N·m), or section modulus (Z, m^3^), but as high-throughput phenotypic proxies whose interpretation can be strengthened through calibration against mechanical ground-truth measurements. Establishing this mechanics–structure–spectral linkage provides the biophysical basis for scaling lodging analysis from plant mechanics to field phenotyping.

### 5.1 UAV platforms and sensor configurations

UAV remote sensing allows crop lodging detection to scale from ground-based point measurements to spatially comprehensive coverage. Multi-rotor UAVs are widely used for fine-scale field monitoring because of their hovering stability and centimeter-level spatial resolution, whereas fixed-wing platforms are better suited to rapid surveys over large areas (Xu et al., 2023). In practice, flight-parameter selection involves a trade-off between survey efficiency and spatial resolution: higher flight altitudes increase ground coverage but reduce spatial detail, requiring application-specific parameter optimization (Long et al., 2025; Wang et al., 2024a).

Red, green, and blue (RGB) cameras are the most widely used UAV sensors because of their low cost and straightforward data interpretation (Zhang et al., 2024b). Lodging detection with RGB imagery relies primarily on changes in canopy texture and visible-light reflectance caused by structural collapse (Liu et al., 2025a). When combined with lightweight semantic-segmentation networks or targeted data-augmentation strategies, RGB imagery can provide robust pixel-level delineation of lodged areas (Zhou et al., 2026b; Zhu et al., 2025). However, the broad and limited spectral bands of RGB cameras are less sensitive to subtle physiological deterioration, which limits their ability to distinguish early or mild lodging (Azizi et al., 2024).

Multispectral sensors extend beyond RGB imaging by adding near-infrared (NIR) and red-edge bands that are highly sensitive to vegetation structure (Wei et al., 2025). Analysis commonly relies on vegetation indices such as the normalized difference vegetation index (NDVI). Canopy collapse reduces multiple scattering, leading to lower NIR reflectance and consequently lower NDVI values, which provide an important spectral indicator of lodging (Kumar et al., 2024). Multispectral sensors offer a practical balance between cost and information content, supporting multi-scale monitoring from UAVs to satellites (Zhang et al., 2026a) as well as reliable estimation of biomass in lodged stands (Zhang et al., 2026c).

Hyperspectral sensors provide much denser spectral sampling, with hundreds of contiguous narrow bands that can capture physiological changes associated with lodging, including pigment degradation and cellular structural damage (Celik et al., 2026; Liao et al., 2024). In time-series growth monitoring and yield estimation under lodging stress, hyperspectral data can outperform multispectral data in some applications (Li et al., 2026c; Wang et al., 2021). Synthetic aperture radar (SAR) provides an important complement to optical remote sensing by exploiting changes in the polarization signatures of canopy volume and surface scattering before and after lodging. SAR can therefore extend monitoring under cloudy or rainy conditions when optical observations are limited (Wang et al., 2026a). However, SAR-based lodging assessment remains sensitive to incidence angle, polarization configuration, speckle, canopy and soil moisture, and geometric effects, and radar backscatter is generally less straightforward to interpret physically than optical reflectance.

**Figure 4** shows that lodging-induced changes in canopy structure remain detectable at satellite scale, demonstrating the feasibility of extending plot-level phenotyping to regional damage assessment. At the same time, the relatively subtle boundaries between lodged and non-lodged areas highlight the need for rigorous radiometric calibration, multi-temporal information, and robust classification algorithms when transferring lodging detection from UAV-scale experiments to operational large-area monitoring.

**Figure 4:**
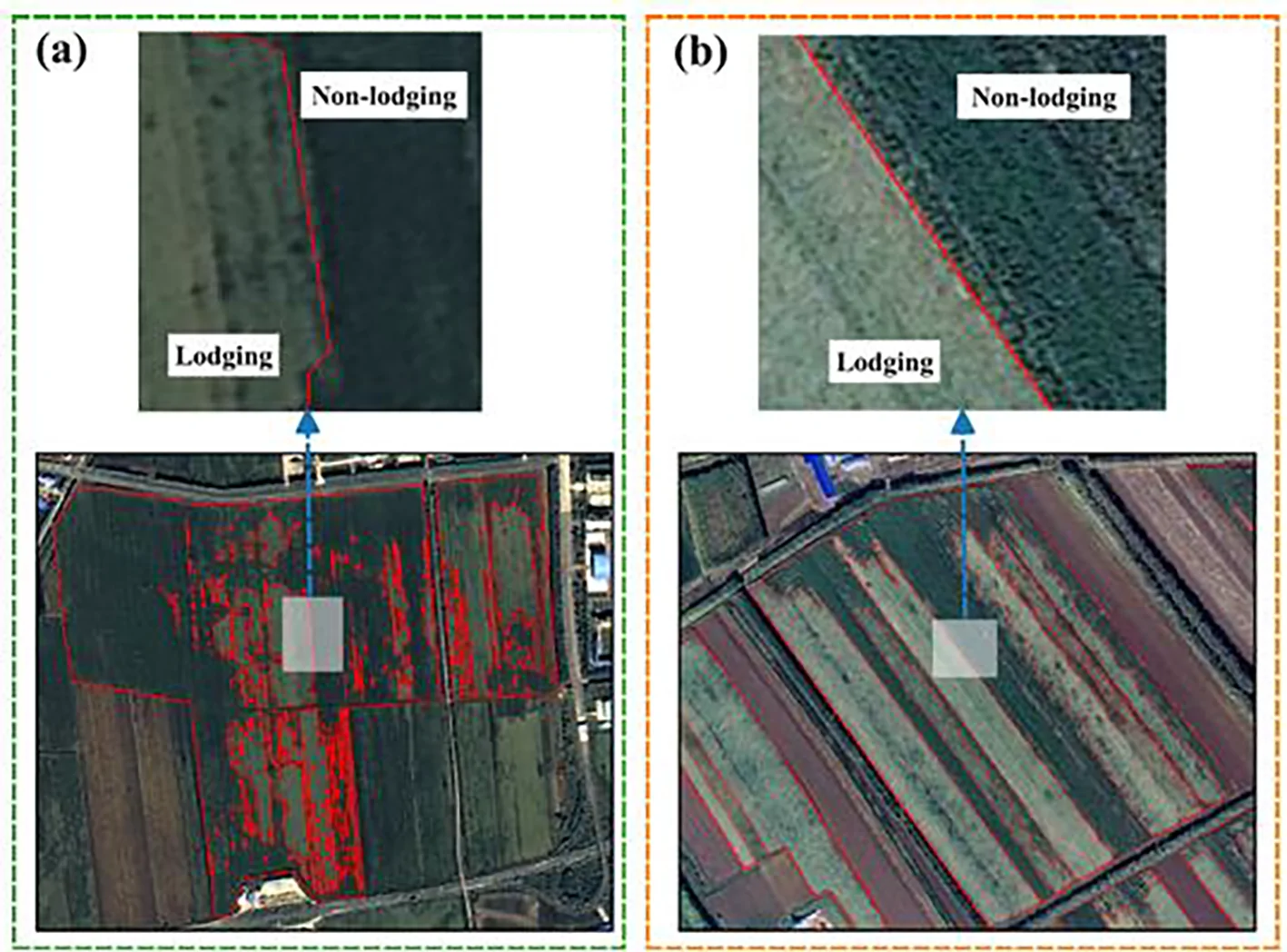
Examples of visually interpreted lodging and non-lodging areas used as reference data for estimating crop lodging percentage from Sentinel-2 imagery. **(a)** Field boundaries and lodged areas overlaid on a GaoFen-1 pan-sharpened image (2 m spatial resolution) acquired on September 23, 2020, in Lindian County, Heilongjiang Province, China. **(b)** Field boundaries and lodged areas overlaid on a Google Earth high-resolution image (0.15 m spatial resolution). In both panels, red lines delineate field boundaries and lodged areas, and the enlarged insets show the boundary between lodging and non-lodging zones. Reproduced from Guan et al. (2022).

UAV sensor selection should therefore be application driven, balancing spatial and spectral resolution against equipment cost and computational complexity (Zhang et al., 2023a). Regardless of platform, rigorous radiometric calibration and geometric mosaicking are essential for cross-temporal and cross-platform comparability (Chen et al., 2026). With the increasing integration of edge-computing hardware, UAV-based lodging detection is shifting from delayed offline processing toward real-time onboard decision-making (Pan et al., 2025).

Ground-based mobile and terrestrial LiDAR systems provide a complementary route for high-throughput lodging phenotyping by acquiring dense three-dimensional canopy information at close range. In spring wheat, an uncrewed ground vehicle equipped with LiDAR was evaluated across six Canadian research stations during the 2023 and 2024 field seasons and achieved robust joint phenotyping of plant height and lodging, demonstrating the value of close-range 3D sensing for breeding-field assessment (Ravichandran et al., 2025). A complementary wheat phenotyping framework combined ground-based LiDAR with 3D point-cloud segmentation to extract plot height, canopy area, canopy volume, plot extension distance, and lodging angle, and then linked these traits to genetic variation through GWAS (Zhou et al., 2026a). Together, these studies indicate that lodging phenotyping is progressing from categorical damage mapping toward quantitative reconstruction of mechanically relevant 3D canopy geometry.

### 5.2 Vegetation indices and spectral features for lodging identification

Building on the mechanics–structure–spectral linkage described above, vegetation indices (VIs) provide quantitative descriptors of lodging-induced optical changes through combinations of specific spectral bands. Among the available spectral regions, the near-infrared plateau (750–900 nm) and red-edge region (680–740 nm) are particularly sensitive to lodging-related changes in canopy structure and physiological status (Derraz et al., 2023; Celik et al., 2026).

Among the available indices, NDVI is widely used because of its computational simplicity and sensitivity to vegetation cover, and a decline in NDVI can serve as an effective indicator of lodging (Fernandez-Jibaja et al., 2025). However, no single index is sufficient under all field conditions, making complementary indices valuable. The green normalized difference vegetation index (GNDVI) is more sensitive to lodging-associated senescence and yellowing, while the normalized difference red-edge index (NDRE) and enhanced vegetation index (EVI) can reduce saturation under high-biomass conditions (Kumar et al., 2024; Li et al., 2026c). The soil-adjusted vegetation index (SAVI) incorporates an adjustment factor that reduces interference from soil and water backgrounds in paddy and dryland fields (Gopinath et al., 2024). Common indices such as NDVI and GNDVI have also supported cross-scale lodging monitoring from UAV imagery to Sentinel-2A satellite observations (Zhang et al., 2026a).

As spectral dimensionality increases, the number of candidate vegetation indices can grow rapidly, creating substantial multicollinearity. Linear classifiers are particularly sensitive to this problem, whereas tree-based ensemble methods are generally more robust and can integrate many correlated high-dimensional indices without stringent spectral pre-filtering (Wei et al., 2025). Robustness can be further improved by combining nadir spectral measurements with multi-angle observations characterized by the bidirectional reflectance distribution function (BRDF), texture features, and SAR polarization indices. SAR is particularly valuable as a complementary source under cloudy or rainy conditions when optical observations are limited (Haq et al., 2025; Yang et al., 2015). Rigorous radiometric calibration and appropriate sensor corrections remain essential for ensuring cross-temporal and cross-platform comparability of time-series vegetation indices (Wang et al., 2024c; Zhu et al., 2024).

Overall, spectral identification of lodging has developed into a progressive methodological framework that combines band selection, complementary vegetation indices, and intelligent fusion of multimodal features. By linking physical optical responses to high-dimensional feature extraction, this framework provides quantitative descriptors for lodging severity assessment and a conceptual basis for the subsequent discussion of spectral discrimination between abiotic- and biotic-associated lodging.

### 5.3 Spectral discrimination between abiotic and biotic lodging

Under field conditions, lodging can arise from two broad classes of causes: abiotic forces such as wind and rain, and biotic stresses associated with pests and diseases such as planthoppers and sheath blight. Although both can appear as canopy collapse in large-scale imagery, their spatial patterns and appropriate interventions differ substantially. Identifying the dominant lodging trigger is therefore important for moving from general damage assessment to targeted agronomic decisions, such as pesticide application, drainage, or crop-straightening interventions (Wang et al., 2024c). However, single-date broadband vegetation indices alone are often insufficient for robust discrimination between abiotic- and biotic-associated lodging. It should be emphasized that most studies demonstrating spectral discrimination between abiotic and biotic lodging have been conducted under simplified conditions in which a single dominant stressor could be identified, and independent validation under mixed-field conditions with confirmed ground truth remains limited.

Under idealized conditions in which a single dominant trigger can be identified, spectral differences between abiotic- and biotic-associated lodging can be interpreted in terms of their distinct contributions to canopy structural and biochemical change. Abiotic lodging is initially dominated by physical canopy deformation, with spectral changes arising mainly from altered 3D structure, leaf orientation, and stem or soil exposure (Cirillo et al., 2025; Sanaeifar et al., 2023). By contrast, lodging associated with pathogens or pests may be preceded or accompanied by biochemical and physiological deterioration. Planthopper infestation, for example, can induce chlorophyll degradation and increase red reflectance (Wang et al., 2024c), whereas sheath blight may impair vascular function and alter water-related spectral responses in the shortwave-infrared region (Sanaeifar et al., 2023). These contrasting pathways provide a mechanistic basis for spectral cause discrimination, but they should not be assumed to produce universally separable signatures under field conditions.

Translating this concept to field application remains methodologically challenging. First, signals can overlap: under dense, high-yield planting conditions, physical lodging may deteriorate the canopy microenvironment and promote secondary pathogen infection, entangling structural and disease-related spectral responses (Wu et al., 2026). Second, phenology can confound diagnosis because normal senescence produces leaf yellowing that is spectrally similar to disease-induced chlorosis, increasing the risk of false-positive classification (Xue et al., 2025). Time-series remote sensing offers a promising way to address both problems by tracking the spectral trajectory of disease stress before lodging and thereby providing temporal evidence for biotic triggers (Wu et al., 2026).

A two-step strategy that first accounts for phenological effects and then extracts stress-related signals can help reduce phenological confounding (Xue et al., 2025). Quantitative inversion of hyperspectral functional traits, including chlorophyll and water content, using radiative transfer models can further provide a more informative basis for multidimensional stress classification (Ren et al., 2026b; Sanaeifar et al., 2023). Deep learning architectures can automatically integrate spectral and spatial features (Li et al., 2025c), while end-to-end multiclass models trained on high-quality semantic-segmentation datasets offer a potential route to trigger identification (Wang et al., 2025b). Future priorities include establishing large, standardized ground-truth datasets that encompass multiple triggers and developing structural–biochemical fusion indices for real-time onboard decision-making and intelligent agronomic diagnosis (Pan et al., 2025; Wang et al., 2021).

Spectral differentiation of abiotic- and biotic-associated lodging should therefore be regarded as an emerging diagnostic framework rather than an established solution for causal attribution. Its robustness still requires validation across cultivars, phenological stages, environments, and mixed-stress conditions using temporally resolved and independently verified ground-truth datasets. Accordingly, the spectral attribution of lodging causes discussed here should be read as a hypothesis-generating framework rather than as a validated diagnostic procedure. With the relevant structural and spectral descriptors defined, the next analytical step is to determine how they can be converted into robust lodging predictions. Section 6 therefore shifts from sensing features to inference algorithms, examining how conventional machine learning and deep learning transform multimodal lodging phenotypes into classification, segmentation, severity assessment, and related diagnostic outputs.

## 6 Machine learning and deep learning for lodging detection

### 6.1 Conventional machine-learning approaches

Conventional machine-learning methods enabled lodging detection to move from empirical thresholding toward data-driven classification. Random forest (RF) and support vector machine (SVM), in particular, established widely used feature-engineering frameworks for this transition (Guan et al., 2022). These methods are well suited to small-to-medium datasets because they can achieve good data efficiency and offer relatively interpretable relationships between engineered features and model outputs. Their main limitation is that feature construction depends heavily on domain expertise and can be labor intensive, while representational capacity is constrained by the predefined descriptors (Zhang et al., 2020a). By contrast, end-to-end deep learning generally benefits from larger annotated datasets, although transfer learning, data augmentation, weak supervision, and task-specific fine-tuning can substantially reduce annotation requirements (Chen et al., 2025a; Guo et al., 2023).

Feature extraction is a critical step in conventional lodging classification because it constrains the information available to the classifier. Input descriptors generally fall into three groups. Spectral features include vegetation indices and band reflectance values that capture changes in canopy reflectance after lodging (Guan et al., 2022; Wu et al., 2019). Texture features derived from gray-level co-occurrence matrices describe canopy disorder caused by intermingled stems and leaves and can provide complementary discrimination when spectral contrast is weak, particularly in early or mild lodging (Rajapaksa et al., 2018). Structural features derived from canopy height models (CHMs) capture the three-dimensional reduction in canopy height caused by lodging (Wilke et al., 2019). Empirical studies show that combining spectral, textural, and structural features generally yields higher classification accuracy than using any single feature group alone (Li et al., 2021).

Different classifiers offer distinct strengths for lodging data. Random forest (RF) is widely used because it handles high-dimensional, correlated features and is relatively insensitive to hyperparameter settings. When radar and optical features are integrated for assessment of typhoon-induced lodging, RF can perform feature selection while maintaining high classification accuracy (Wang et al., 2020). Support vector machines (SVMs), by contrast, use kernel functions to map data into higher-dimensional spaces and can delineate nonlinear class boundaries more effectively than linear discriminant models in complex lodging-severity classification (Guo et al., 2023). Overall, tree-based ensemble methods are often robust when lodging datasets combine high feature dimensionality with limited sample size.

Conventional machine-learning methods remain particularly valuable for satellite-based lodging detection, where their relatively low data requirements and ability to integrate heterogeneous features are well suited to large-area monitoring. For regional damage assessment, temporal change analysis using multi-temporal Sentinel-2 or high-resolution satellite imagery shifts lodging detection from single-date spatial classification toward change-based identification, as demonstrated for maize using high-resolution satellite imagery (Chen et al., 2022), thereby reducing interference from relatively stable background conditions and phenological variability (Guo et al., 2023). This approach was demonstrated in maize, but the underlying principle of change-based detection is transferable to rice and wheat and is therefore discussed here as a methodological reference rather than as direct evidence for these two crops. When cloud cover and precipitation limit the availability of optical imagery, radar backscattering features can further complement optical observations and support more robust all-weather lodging assessment (Wang et al., 2020).

Nevertheless, conventional machine-learning approaches remain constrained by their dependence on manually engineered features. Predefined spectral, textural, and structural descriptors may not fully capture the complex and spatially heterogeneous manifestations of lodging, while conventional pixel-, object-, or patch-based classifiers generally have limited capacity to learn hierarchical representations and contextual relationships directly from imagery. These limitations can reduce model transferability across cultivars, growth stages, and ecological environments. Such challenges have motivated the transition toward end-to-end deep-learning approaches, particularly convolutional neural network-based semantic segmentation, which jointly learn multiscale image representations and spatial context for pixel-level lodging delineation, as discussed in Section 6.2.

### 6.2 Deep learning for pixel-level semantic segmentation

Deep learning-based semantic segmentation has shifted lodging detection from manual feature engineering toward automated representation learning (Wang and Xiao, 2024; Zhang et al., 2024b). Convolutional neural network (CNN) encoder–decoder architectures learn hierarchical visual features directly from raw imagery and generate pixel-level lodging masks (Zhang et al., 2024a). This dense-prediction paradigm reduces the loss of spatial context associated with traditional grid- or patch-based classification and provides a direct basis for damage-area estimation and spatial mapping (Kumar et al., 2024; Long et al., 2025). U-Net and related architectures are particularly influential because skip connections combine fine spatial detail with higher-level semantic information. Their ability to delineate irregular and gradually changing lodging boundaries has made U-Net a widely adopted backbone for crop-lodging segmentation (Su et al., 2022; Zhang et al., 2024a).

**Figure 5** demonstrates that 3D point-cloud reconstruction can move lodging phenotyping beyond binary area segmentation toward the quantitative extraction of structural traits, including plant height, canopy geometry, and lodging angle. These geometrically interpretable traits provide an important intermediate representation for linking image-based detection with the mechanical deformation states discussed in Section 4.2.

**Figure 5:**
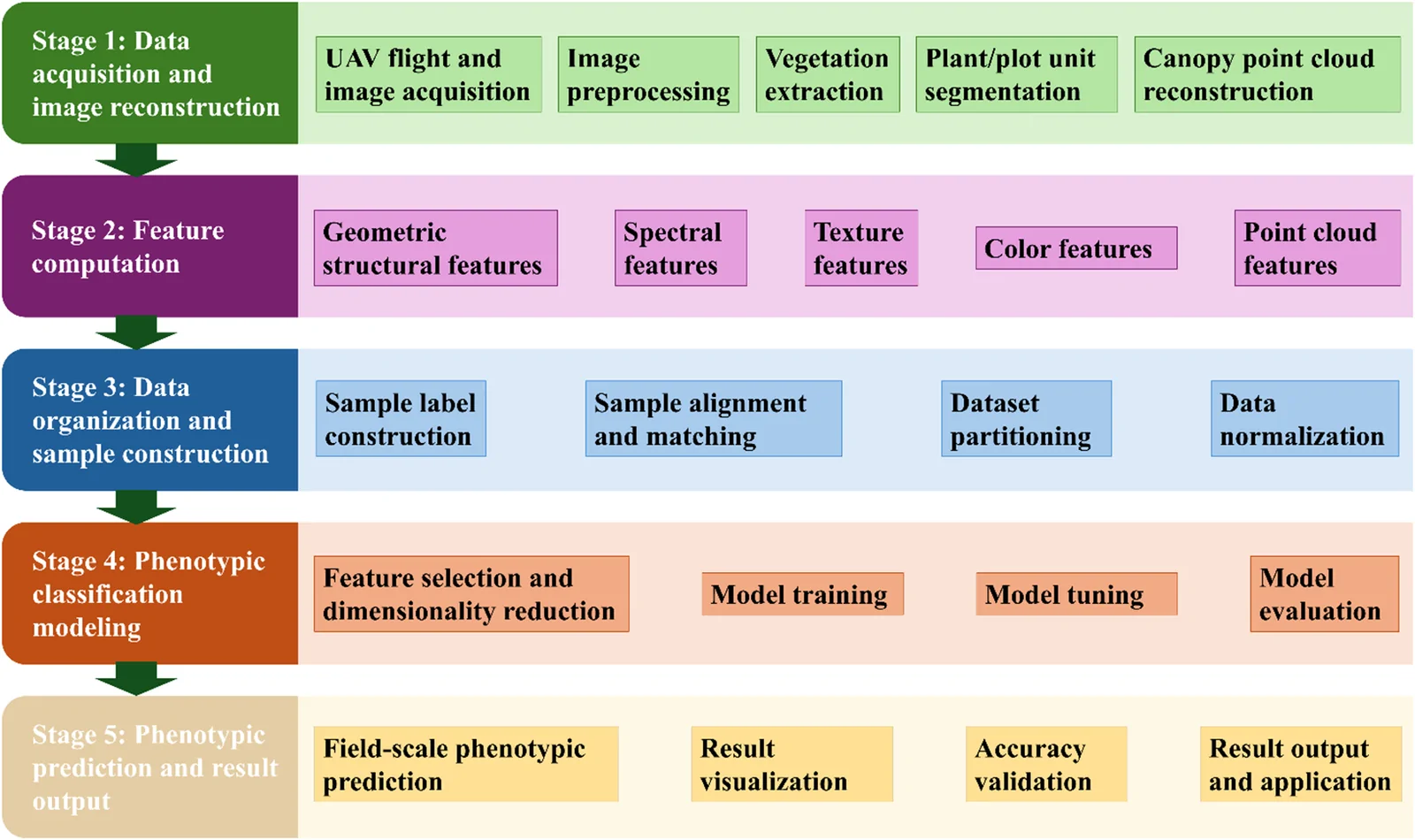
Integrated workflow for lodging phenotyping and detection using multi-source sensing data. The workflow includes multi-source data acquisition, feature extraction, sample construction and preprocessing, model development and evaluation, and visualization and validation of lodging detection results.

To address diverse and complex field conditions, numerous specialized architectures have been developed from the U-Net framework. LodgeNet uses attention mechanisms to strengthen feature responses in lodged areas (Su et al., 2022); SegNeXt-RCMSCA combines multiscale channel attention to detect both small patches and extensive lodging (Guo et al., 2025); and AAUConvNeXt uses modern convolutional designs to balance detection accuracy and computational efficiency (Zhang et al., 2024a). Field deployment nevertheless involves several engineering trade-offs. UAV flight altitude must balance spatial resolution at low altitude against greater coverage at high altitude (Long et al., 2025). For edge deployment, lightweight networks such as WLUSNet use depthwise separable convolutions to reduce parameter count and support real-time inference (Zhu et al., 2025). For ultra-high-resolution imagery, overlapping grid-based inference can reduce GPU memory requirements while preserving segmentation coverage (Wang and Xiao, 2024). At the microscopic scale, deep learning has also been combined with micro-CT imaging for 3D segmentation and quantitative extraction of stem anatomical traits related to lodging resistance (Wu et al., 2021), illustrating how automated image analysis can convert labor-intensive structural measurements into scalable digital phenotypes.

Despite its high pixel-level accuracy, semantic segmentation still faces four major barriers to large-scale application. First, pixel-wise mask annotation is labor intensive and limits the efficient construction of large natural-scene datasets (Chen et al., 2026). Second, spatial class imbalance can bias model predictions and often requires tailored loss functions (Guo et al., 2025). Third, domain shift caused by differences in canopy structure, illumination, or environment can substantially reduce performance when models are transferred to new regions (Zhu et al., 2025). Fourth, end-to-end models often provide limited agronomic and physical interpretability (Kumar et al., 2024; Wu et al., 2021). These limitations are motivating a transition from purely data-driven segmentation toward multi-task and mechanism-constrained learning that incorporates auxiliary objectives and physical prior knowledge. Viewed collectively, these architectures are not merely classifiers that improve pixel-level accuracy; they learn hierarchical visual representations that correspond to biologically interpretable lodging patterns, ranging from edge-level canopy texture to region-level spatial continuity of lodged patches. When combined with three-dimensional sensing, the same representations yield quantitative traits such as lodging angle, canopy height reduction, and patch geometry, which can be directly related to the mechanical deformation states described in Section 4.2.

### 6.3 Mechanism-constrained, multi-task, and edge-based real-time inference

Although purely data-driven semantic-segmentation models perform well in pixel-level feature extraction, their single-task architectures and opaque decision processes limit broader use in precision agriculture. Multi-task learning, mechanism- or rule-constrained learning, and edge computing represent complementary directions for overcoming these limitations by integrating data with prior knowledge and moving inference from offline post-processing toward onboard real-time decision-making. Each of these directions is therefore evaluated below not only in terms of computational performance but also in terms of the biological and biophysical information it can deliver.

Multi-task learning jointly optimizes related objectives through shared representations, allowing complementary information to improve model generalization. In agronomic applications, this framework has been used for joint lodging detection and severity grading (Chauhan et al., 2020) and for combined lodging detection and yield-loss estimation (Wei et al., 2025). Cross-domain transfer learning can further reuse feature extractors pre-trained on large natural-image datasets for crop-specific tasks, reducing the need for extensively annotated target-domain data (Wei et al., 2025; Zhang et al., 2020a).

Rule-constrained learning seeks to reduce physically inconsistent outputs from purely data-driven models by incorporating agronomic prior knowledge—such as canopy geometry and mechanical lodging mechanisms—into network inductive biases or loss-function regularization (Zhang et al., 2026b). For example, the boundary-aware optimization strategy in Lodge-Unet incorporates the prior that lodging-patch boundaries should be spatially continuous, improving segmentation along transition zones (Liu et al., 2025a). Spatial self-attention can likewise adaptively emphasize local neighborhood context and thereby impose a soft spatial constraint on model predictions (Zhang et al., 2026b). Directional information such as stem tilt azimuth has also been incorporated into decision pipelines, extending lodging detection toward path planning for adaptive, low-loss harvesting (Xie et al., 2023).

Edge computing has accelerated the deployment of lodging assessment from remote or cloud-based processing to onboard UAV platforms, supporting rapid decisions for post-disaster management and adaptive machinery operation (Pan et al., 2025; Yang et al., 2020). Model quantization, channel pruning, and architectural simplification can reduce computational requirements sufficiently to achieve millisecond-scale inference on embedded hardware (Gao et al., 2023; Pan et al., 2025). Such algorithm–hardware co-optimization has enabled autonomous UAV systems that integrate flight, online detection, and real-time prescription generation (Yang et al., 2020). Under dense canopies or when optical sensing is unreliable, adaptive image segmentation can also be complemented by force-sensor-based tactile recognition near the crop–machine interaction zone (Chen et al., 2025b).

In summary, multi-task learning integrates complementary agronomic information, rule-constrained learning introduces physical and agronomic priors, and edge computing enables real-time field deployment. Together, these approaches are moving lodging detection from purely data-driven analysis toward knowledge-guided, field-deployable intelligent perception. Future directions include physics-informed neural networks that explicitly incorporate lodging mechanics and digital-twin decision-support systems capable of integrating internal crop states with external management responses (Yang et al., 2023).

To facilitate quantitative comparison across representative machine-learning and deep-learning studies, **Table 1** summarizes differences in sensing modality, dataset scale, lodging classes, validation strategy, model performance, external validation, computational efficiency, and major limitations.

**Table 1:** Quantitative comparison of representative machine-learning and deep-learning approaches for crop lodging detection.

Study	Crop/sensor	Dataset scale	Lodging classes/task	Validation strategy	Model	Reported performance	External validation	Speed/hardware	Main limitation
Guan et al. (2022)	Crop lodging; Sentinel-2	1462.62 ha across six counties/districts	Continuous lodging percentage	Train–test evaluation of aggregated Sentinel-2 grids	RF regression	R^2^ = 0.64; RMSE = 25.24%	Preliminary regional validation: mapping across six administrative areas; independent-year validation not reported	NR	Moderate predictive accuracy; mixed-pixel and satellite-resolution effects
Kumar et al. (2024)	Rice; 10-band UAV multispectral	Single experimental farm; plot-level samples	Lodged vs. standing	RF classification; lodging maps validated against visually interpreted RGB reference	RF	OA = 96.1%; κ = 0.92; lodging-area validation R^2^ = 0.97	Not reported beyond the same experimental site	NR	Single-site dataset; transfer across varieties/regions not tested
Su et al. (2022)	Rice; high-resolution UAV imagery	Small self-built dataset; exact sample number not clearly reported	Lodging semantic segmentation	Internal training/test evaluation	LodgeNet	Accuracy = 97.30%	Not reported; internal evaluation only	NR	Small dataset; cross-site/year/cultivar generalization not demonstrated
Sun et al. (2023)	Rice; RealSense D435/onboard RGB	Self-built field dataset; exact sample number not clearly reported	Lodged vs. non-lodged segmentation	Internal model evaluation + harvester application test	RL-DeepLabv3+	mIoU = 90.52%; mPA = 94.73%	Preliminary field deployment; independent site/year validation not reported	50 FPS at 640×480; 21 FPS at 1280×720; onboard IPC	Dataset and environmental diversity limited
Zhang et al. (2024a)	Rice; UAV RGB	3,941 image patches (512×512) derived from eight orthomosaics	BG, non-lodged, half-lodged, lodged	75:25 internal train/validation split	AAUConvNeXt	PA = 96.3%; mPA = 96.3%; mIoU = 93.2%	Not reported beyond the same experimental area	RTX 4090 during experiments	Strong internal performance, but independent field/cultivar transfer not tested
Long et al. (2025)	Wheat; UAV RGB at 15, 45 and 91 m	Plot datasets generated from one wheat experiment	Lodging angle, location and ratio	Comparison across three UAV flight heights	MobileNetV3-M	Mean Acc.: angle 86.8%, location 86.4%, ratio 84.4%; at 15 m: 89.7%, 89.8%, 88.8%	Not reported beyond a single site and wheat variety	NR	Authors explicitly identify single variety/site as a generalization limitation
Zhu et al. (2025)	Wheat; UAV RGB	Self-built multi-scene UAV dataset	Lodged vs. non-lodged segmentation	Internal evaluation + generalization experiment	WLUSNet	mIoU = 86.90%; mPA = 93.26%	Preliminary generalization test; broad multi-year/multi-location validation not reported	26.94 FPS; model size 4.1 M	External validation remains limited despite multi-scene testing
Yao et al. (2025)	Rice; RealSense depth/RGB on combine harvester	Self-built field dataset	Lodged vs. non-lodged + crop height	Internal segmentation evaluation + onboard harvesting-field test	Modified DeepLab V3+	Lodged: PA = 93.83%, mIoU = 85.30%; non-lodged: PA = 93.77%, mIoU = 83.97%; height error<4%	Preliminary harvester field deployment; independent region/year validation not reported	Jetson Nano + ROS	Harvester-specific configuration; broader environmental transfer not established

Acc., accuracy; BG, background; FPS, frames per second; ha, hectare; IPC, industrial personal computer; κ, Cohen’s kappa coefficient; M, million; mIoU, mean intersection over union; mPA, mean pixel accuracy; NR, not reported; OA, overall accuracy; PA, pixel accuracy; R^2^, coefficient of determination; RF, random forest; RGB, red, green, and blue; RL, reinforcement learning; RMSE, root mean square error; ROS, Robot Operating System; RTX, NVIDIA GeForce RTX. “External validation” refers here to evaluation using data independent of the primary training environment, such as different fields, years, cultivars, or geographic locations. Field deployment using the same experimental population is therefore described as preliminary rather than as full external validation.

Despite the strong within-dataset performance reported for many lodging-detection models, **Table 1** shows that external validation remains less common than internal evaluation. Most studies rely on random or internal partitioning of data from a single site, season, or cultivar, whereas independent cross-site, cross-year, cross-cultivar, and cross-location evaluations remain uncommon. It should be emphasized that the numerical values listed in **Table 1** are not directly comparable across studies, because sensor configuration, spatial and spectral resolution, lodging definition, plot size, phenological stage, dataset composition, and evaluation metrics differ substantially among them. The table is therefore intended to illustrate the diversity of methodological approaches rather than to rank models, and any comparison of reported accuracy across entries should be made with these methodological differences in mind. Future benchmarking should prioritize standardized external test sets and explicitly report domain-transfer performance in addition to within-dataset accuracy.

## 7 Intelligent sensing and adaptive harvesting automation

Sections 5 and 6 establish how lodging can be observed and recognized from field-scale data; practical harvesting, however, requires these phenotypes to be converted into machine actions in real time. At the engineering level, this transition follows a perception–control–navigation hierarchy. Real-time lodging sensing first estimates spatial distribution, severity, canopy height, and stem orientation. Adaptive operation control then converts these estimates into adjustments of harvesting components, and autonomous navigation uses the same spatial information to determine machine motion through heterogeneous fields. The following subsections are therefore organized according to this sensing-to-action chain. The engineering developments reviewed below are drawn from existing studies, but complete closed-loop systems for lodged-crop harvesting have not yet been field-deployed; where this is the case, the discussion is explicitly framed as an emerging concept or a future research direction rather than an established technology.

### 7.1 Real-time lodging sensing

Mechanized harvesting of lodged crops has long been constrained by the lack of real-time lodging sensing on combine harvesters (Liu et al., 2026). Conventional operations rely heavily on visual observation by the operator to adjust header height and reel position, making them vulnerable to errors under severe lodging or poor illumination (Wen et al., 2022). To address this limitation, vehicle-mounted systems now combine multimodal sensors, including depth cameras and light detection and ranging (LiDAR), with lightweight edge-deployed deep-learning models to convert qualitative lodging observations into quantitative real-time measurements. These measurements provide the environmental information required for subsequent adaptive control (Gao et al., 2023). Current approaches primarily use vision-based regional recognition and LiDAR-based 3D reconstruction (Cong et al., 2022; Zhang et al., 2024a).

**Figure 6** illustrates the transition from aerial or offline lodging assessment to vehicle-mounted real-time perception during harvesting. By integrating forward-looking imaging sensors with embedded computing hardware, such systems can convert crop posture and lodging information into onboard decision variables that subsequently support adaptive header control, reel regulation, and travel-path adjustment.

**Figure 6:**
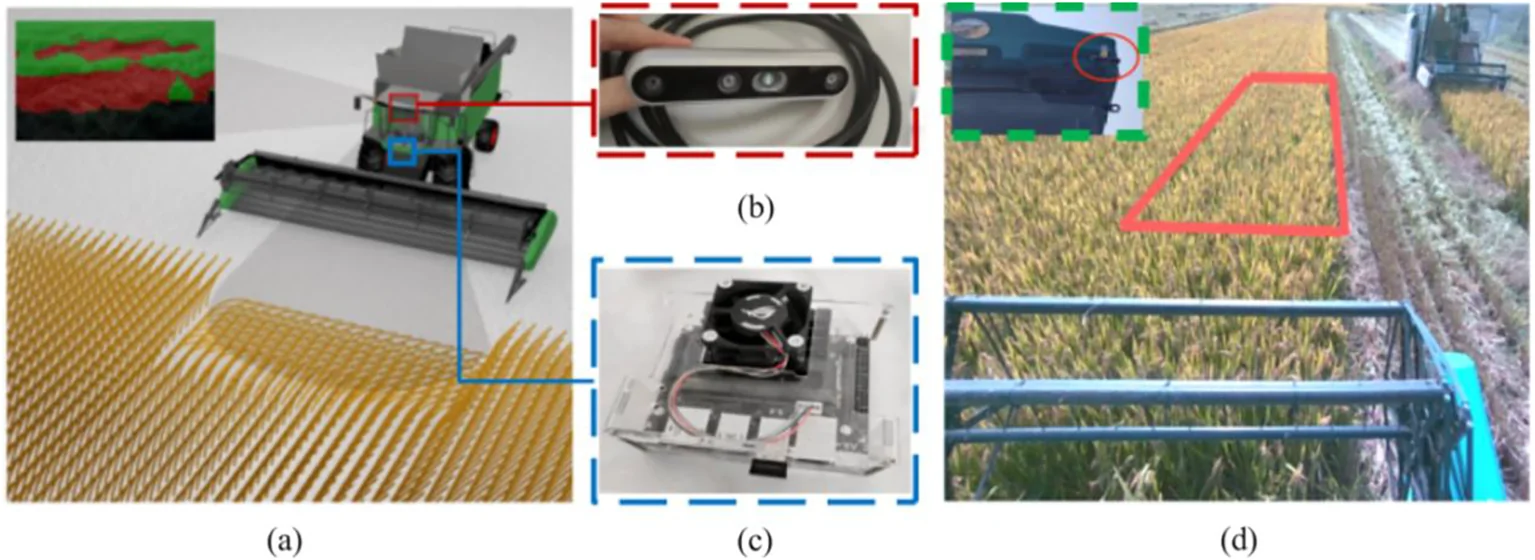
Machine vision system for real-time crop lodging recognition on a combine harvester. **(a)** Schematic diagram of the overall system architecture, showing the workflow from image acquisition using an Intel RealSense D455 depth camera to onboard processing with a Jetson Nano development board and generation of the lodging-area mask and crop-height information. The red and blue dashed boxes correspond to the D455 depth camera and Jetson Nano development board shown in **(b)** and **(c)**, respectively. **(b)** Intel RealSense D455 stereo depth camera used as the primary vision sensor, with a measurement range of up to 6 m, a depth error of<2%, and an image resolution of 640 × 480 pixels. **(c)** NVIDIA Jetson Nano development board used as the onboard computing unit for semantic segmentation (DeepLab V3+) and depth-data processing. **(d)** Perspective view of the combine harvester operating in the field. The green dashed box identifies the enlarged inset showing the camera installation position on the harvester cabin, with the camera position indicated by the colored circle. The red outlined region in front of the harvester denotes the recognition target area, i.e., the region of interest used for real-time lodging detection and crop-height measurement. The camera was mounted approximately 2.7 m above the ground with a downward tilt angle of 30° to capture the forward harvesting area. Reproduced from Yao et al. (2025).

Depth cameras and binocular vision are widely used because they offer moderate cost and integrate readily with deep-learning models. Lightweight CNNs can perform real-time lodging recognition on embedded platforms. For example, binocular wheat-canopy imagery combined with semantic-segmentation networks enables pixel-level discrimination of lodged regions during harvesting (Wen et al., 2022), while clustering-based extraction of stem tilt direction provides decision variables for selecting favorable harvesting directions (Zhang et al., 2023c). To meet the computational constraints of onboard edge devices, reinforcement-learning-based neural architecture search has substantially reduced the parameter count of rice-lodging segmentation models (Sun et al., 2023). Forward-looking vision systems with dynamic posture correction have also enabled real-time crop-height estimation, providing stable feedforward signals for adaptive header-height control (Liang et al., 2026b).

Because no single sensing modality simultaneously maximizes measurement accuracy, environmental robustness, computational efficiency, and affordability, sensor selection for lodged-crop harvesting requires explicit engineering trade-offs. **Table 2** therefore compares the major vehicle-mounted sensing technologies according to their data outputs, field adaptability, cost, and reported performance.

**Table 2:** Comparison of major vehicle-mounted sensing technologies for lodged-crop harvesting.

Sensor type	Output data	Advantages	Field adaptability	Relative hardware cost	Key performance	Reference
Stereo Camera (e.g. Intel RealSense D435/D455)	Disparity map, depth map, RGB image (2560 × 720 at 60 FPS)	Low cost and simple structure; mature SGM algorithm; rich scene information; easy DL integration; less shadow-sensitive vs. monocular	Static precision ~5 mm; affected by strong light and vibration; point cloud density decreases with distance	Low	D455: range 0–6 m, depth error<2%; D435: FOV 87° horizontal × 58° vertical, max range 10 m; built-in 200 Hz IMU; stereo+ToF fusion robust under variable light and dust	(Liang et al., 2026b; Wen et al., 2022; Zhang et al., 2023c)
ToF Camera (e.g. Kinect v2, PMD CamBoard)	Depth image (per-pixel distance), RGB-D, point cloud	Direct distance output; active IR illumination (works in darkness); no feature matching; fast processing	Susceptible to strong sunlight (IR saturation); performance degrades in dense canopy/occlusion; effective range ~0.5–5 m (consumer-grade)	Moderate	Processing latency ~24 ms; canopy grid mapping for navigation; fused with stereo for robustness under dust and variable lighting	(Liang et al., 2026b; Liu et al., 2025a; Xie et al., 2023; Yao et al., 2025)
LiDAR (e.g. Velodyne, Ouster, Livox)	3D point cloud (x, y, z, intensity), range image, reflectance	Highest precision (cm-level); strong light robustness; penetrates vegetation to ground; rich 3D structural information	High cost limits wide adoption; heavy point cloud processing; channel-limited angular resolution; occlusion issues	High	LiDAR/IMU/GNSS fusion: cm-level positioning, average lateral offset = 2 cm; lodging detection error<9 cm/<9°	(Liang et al., 2026b; Xie et al., 2023; Yao et al., 2025)
IMU (accelerometer + gyroscope)	3-axis acceleration, 3-axis angular velocity, attitude (Roll/Pitch/Yaw)	Very low cost; no external reference signal required; high update rate (200–400 Hz); small form factor	Cumulative drift over time, so absolute positioning requires fusion with GNSS or vision; affected by vibration and terrain	Low	RealSense D455: IMU at 200 Hz; IMU with dynamic correction: crop height RMSE reduced by 43% (MAPE reduced by 47%), cutting width MAE reduced by 28%; IMU with 3-line laser guidance: ~1 cm accuracy	(Liang et al., 2026b; Sun et al., 2022; Yao et al., 2025)
Ultrasonic Sensor	Distance (1D range), acoustic ToF	Very low cost; simple interface; lighting-independent; low power	Signal reflection errors in dense vegetation; stability degrades in complex fields with obstacles; limited resolution	Low	Limitations: dense vegetation causes reflection errors; obstacles degrade performance; suitable only for simple profiling tasks	(Liu et al., 2026; Shan, 2025)
Monocular Camera (e.g. CCD/CMOS)	RGB image (2D only; no direct depth)	Simplest structure; lowest cost and power; mature algorithms (DL-ready)	Limited depth information (needs auxiliary algorithms); strongly affected by lighting changes; no native 3D data	Low	USB camera + wavelet transform: lodging detection under varying light; RGB + multispectral: higher accuracy via transfer learning	(Zhang et al., 2023c; Zhou et al., 2026b; Zhu et al., 2025)
Multi-Sensor Fusion (Stereo+ToF+IMU+GNSS)	Fused depth map + RGB + attitude + global position (RGB-D-IMU-GNSS)	Complementary strengths; robust to individual sensor failures; potentially improved accuracy in complex conditions	Higher system complexity and cost; requires strict synchronization and calibration; fusion algorithm overhead	Moderate to High	LiDAR+IMU+GNSS: cm-level positioning, average offset 2 cm; Vision+IMU+Encoder: robust crop row tracking; Stereo+ToF: stable point cloud, lodging boundary error<10 cm	(Liang et al., 2026b; Liu et al., 2025a; Yao et al., 2025)

1D, one-dimensional; 2D, two-dimensional; 3D, three-dimensional; CCD, charge-coupled device; CMOS, complementary metal–oxide–semiconductor; DL, deep learning; FOV, field of view; FPS, frames per second; GNSS, global navigation satellite system; IMU, inertial measurement unit; IR, infrared; LiDAR, light detection and ranging; MAE, mean absolute error; MAPE, mean absolute percentage error; RGB, red, green, and blue; RGB-D, red, green, and blue plus depth; RMSE, root mean square error; SGM, semi-global matching; ToF, time of flight; USB, universal serial bus. Cost categories (low, moderate, and high) indicate a qualitative relative ranking of typical research-grade sensor acquisition costs and exclude computing hardware, integration, calibration, and maintenance costs.

Rather than identifying a universally optimal sensor, **Table 2** highlights application-dependent selection. Stereo vision offers a favorable balance between cost and information richness, while LiDAR provides high-quality 3D structural measurements with greater robustness to illumination variability but at a higher cost. Multi-sensor fusion can further improve sensing reliability under vibration, dust, occlusion, and variable illumination, although it requires more complex synchronization and calibration as well as greater computational resources.

LiDAR provides complementary structural information by directly acquiring 3D point clouds and is less sensitive to ambient illumination than passive RGB imaging. Ground-based LiDAR has been used to quantify wheat canopy height, three-dimensional structure, and lodging angle, demonstrating its value for structural crop phenotyping (Zhou et al., 2026a). Such measurements can provide structural references for adaptive header control. Because individual sensors have inherent blind spots in complex harvesting environments, multi-sensor fusion combined with edge computing offers an important route to more robust perception (Sun et al., 2022). For example, spatiotemporal alignment of depth-camera and inertial measurement unit (IMU) data enables accurate online detection of lodged areas (Yao et al., 2025), while low-bit-width quantization on edge platforms can substantially reduce inference latency for real-time rice-lodging assessment (Gao et al., 2023). Specialized networks can further balance segmentation accuracy and inference speed through dual-frequency feature fusion and boundary-aware optimization (Liu et al., 2025a).

Across these sensing modalities, the key outputs of vehicle-mounted lodging perception fall into four categories: 2D spatial masks of lodging boundaries (Zhang et al., 2020a), directional vectors describing stem tilt azimuth (Zhang et al., 2023c), lodging-severity grades (Liu et al., 2026), and continuous canopy-height profiles along the harvester travel direction (Liang et al., 2026b). The accuracy and update rate of these outputs directly affect adaptive-control performance. Major challenges include maintaining stable multimodal sensing under severe vibration and dusty field conditions and further reducing end-to-end latency in the perception–control loop (Yao et al., 2025). Addressing these engineering challenges requires close integration of agricultural equipment and computer-vision research. More importantly, sensing outputs become operationally useful only when they are translated into actuator commands, motivating the adaptive-control layer discussed next.

### 7.2 Adaptive operation control

The main engineering challenges in harvesting lodged crops arise from abrupt reductions in canopy height, disordered stem orientations, and severe plant tangling. These conditions require key combine-harvester components to support dynamic adjustment (Shan, 2025; Xie et al., 2023). Manual hydraulic operation is vulnerable to header ground contact and missed cutting because of operator blind spots and delayed responses (Niu et al., 2025). Adaptive operation control addresses these limitations by using lodging parameters from vehicle-mounted sensing systems to close the loop between perception and actuation. Header height, reel posture, and forward speed can then be adjusted dynamically to reduce harvesting losses under variable field conditions (Li et al., 2022; Niu et al., 2025; Shan, 2025).

Because the header is the first working component to contact the crop, adaptive regulation of its height and attitude is central to low-loss harvesting under spatially variable conditions (Niu et al., 2025; Shan, 2025). Incorporating vision-derived canopy-height feedforward signals into conventional hydraulic systems with proportional–integral–derivative (PID) feedback control can substantially reduce header-height tracking error under lodging conditions, thereby avoiding both excessive stubble height and ground contact (Niu et al., 2025; Shan, 2025). At the control-algorithm level, classical PID performs well under smoothly varying conditions, whereas Model Predictive Control (MPC) can provide better dynamic tracking when lodging severity changes abruptly by optimizing system responses over a moving prediction horizon (Zhao et al., 2025a).

Low-loss harvesting also depends on coordinated regulation of multiple working components rather than header control alone (Xie et al., 2023). Low-lying lodged stems require the reel to operate at a lower position and often at a higher rotational speed to improve crop lifting, but excessive reel speed can increase grain-shattering losses. Reel position and speed must therefore be optimized jointly with header height and forward speed (Zhao et al., 2025a). Studies show that incorporating sensing variables such as lodging direction, lodged-area ratio, and severity into multi-objective optimization can dynamically identify suitable parameter combinations and reduce total harvesting losses (Xie et al., 2023). Because lodged crops can also carry more soil and impurities into the machine, real-time adjustment of cleaning-fan speed based on impurity feedback is important for maintaining grain cleanliness. These examples illustrate the value of coordinated regulation across agronomic requirements and machine parameters (Wu et al., 2022).

Industrial deployment of adaptive harvesting control still faces three major challenges. First, control models must balance precision against robustness. High-fidelity mechanistic models can be difficult to parameterize online, whereas simplified linear models may not capture nonlinear reaction-force changes from soil and crop interactions. Gray-box approaches that combine mechanistic structure with data-driven estimation offer a promising compromise. Second, coordinated control of multiple actuators creates a substantial computational burden. Strong coupling among header, reel, forward-speed, and cleaning-system variables makes full-dimensional real-time optimization expensive, motivating hierarchical or distributed control architectures. Third, standardized methods for evaluating control performance are still lacking. A unified benchmarking framework is needed to move the field from empirical tuning toward rigorous engineering design. Beyond regulating harvesting components, the same real-time lodging information must also guide the spatial motion of the combine harvester, including travel direction, trajectory, and local path selection. Although individual components of adaptive control have been demonstrated in isolation, fully integrated perception–control systems for lodged-crop harvesting remain at an early stage and are therefore discussed here as an emerging direction rather than as established practice. The navigation layer is discussed in the following section.

### 7.3 Autonomous driving and navigation

Autonomous navigation constitutes the third functional layer of the perception–control–navigation chain for intelligent harvesting. Unlike lodging sensing, which determines where and how severely crops are lodged, and adaptive operation control, which regulates working components such as the header and reel, the navigation system determines how the combine harvester should move through spatially heterogeneous fields. Reliable navigation therefore requires the integration of global positioning, local environmental perception, path planning, and trajectory tracking. In agricultural machinery, global navigation satellite system (GNSS)-based positioning is commonly complemented by inertial measurement units (IMUs), cameras, LiDAR, and other onboard sensors to improve localization robustness when satellite signals or visual features become unreliable. Vision- and LiDAR-based crop-row or field-boundary detection further provides local geometric constraints that enable autonomous vehicles to maintain stable travel paths within crop stands (Shi et al., 2023; Ma et al., 2025; Liu et al., 2025b).

Lodged fields impose additional requirements on navigation because the optimal harvesting direction is no longer determined solely by pre-defined crop rows or geometric field boundaries. Spatial variation in lodging direction, severity, and canopy height can generate locally different harvesting conditions, making a fixed travel path suboptimal. The directional information produced by onboard lodging sensing can therefore be incorporated into local path planning. For example, stem tilt azimuth extracted from machine-vision observations can provide a decision variable for selecting harvesting directions that are more favorable for crop lifting and feeding (Zhang et al., 2023c). Multi-feature lodging information has further been used to support adaptive low-loss harvesting decisions, demonstrating the potential for coupling lodging maps and directional traits with local navigation planning (Xie et al., 2023). In this framework, field boundaries and crop rows define the global feasible region, whereas local lodging direction and severity modify the preferred travel direction within that region.

After a feasible path has been generated, accurate trajectory tracking is required to maintain the harvester on the planned route despite wheel slip, uneven terrain, vibration, and rapidly changing crop resistance. Path-tracking approaches developed for autonomous agricultural machinery, including sliding-mode and line-of-sight-based control strategies, provide transferable solutions for maintaining lateral accuracy under field disturbances (Li et al., 2023; Song et al., 2026). Obstacle detection and avoidance must also be incorporated into the navigation layer because lodged crops can partially obscure field obstacles and reduce the reliability of conventional visual cues. Vision-based crop and obstacle perception, combined with local replanning, provides a practical route toward maintaining safe autonomous operation in such unstructured field environments (Gunturu et al., 2022). At headlands, navigation additionally requires coordinated turning and re-entry into the next harvesting swath so that path continuity is maintained without excessive unharvested areas or repeated crop traversal.

The goal is not to treat navigation, sensing, and harvesting control as independent subsystems, but to integrate them within a perception–decision–execution architecture. Real-time lodging maps, canopy-height profiles, and stem-orientation vectors generated by the sensing layer can inform both local path planning and working-parameter adjustment. A transition from upright to severely lodged crop, for example, may require the navigation layer to alter travel direction while the control layer simultaneously lowers the header, adjusts reel operation, and reduces forward speed. Coordinating these responses allows navigation and harvesting-component regulation to act on the same spatial lodging information. A future integrated harvesting workflow could begin with a UAV pre-scan that generates a field-scale map of lodging severity and dominant stem orientation. After transfer to the combine’s onboard computer, this prior map could be fused with real-time vehicle-mounted sensing to plan the harvesting sequence and locally preferred travel direction and to pre-adjust header height, reel posture, and forward speed before the machine enters a lodged patch. Such feedforward coordination would shift harvesting from reactive correction toward proactive perception–decision–execution control. It should be noted, however, that this integrated workflow has not yet been demonstrated end-to-end under field conditions and is presented here as a proposed system architecture. Future work should therefore prioritize several areas. These include robust multi-sensor localization under dust, vibration, canopy occlusion, and intermittent GNSS, real-time local replanning in response to spatially variable lodging, reliable obstacle avoidance and headland turning, and standardized evaluation of navigation accuracy, computational latency, and harvesting loss under realistic lodged-field conditions.

## 8 Conclusion and future perspectives

### 8.1 Core findings

This review establishes a mechanically consistent, cross-scale framework that connects biological regulation to quantifiable engineering parameters and links those parameters to field-observable phenotypes and harvestability. The explicit unification of plant biology, mechanics, sensing, and intelligent harvesting within a common physical language constitutes the central contribution of the review. Within this framework, four biological and biomechanical findings are particularly important. First, the typological categorization into root lodging, stem bending, and stem breaking provides a basis for interpreting distinct mechanical failure mechanisms and for designing differentiated breeding and cultivation strategies, moving beyond treatment of lodging as a single undifferentiated trait. Second, stem lodging resistance is dynamically determined by stem morphology, tissue anatomy, and secondary-cell-wall composition from internode development through grain filling; these traits are functionally coupled through cell-wall assembly processes. Third, genetic improvement of lodging resistance has moved beyond reliance on individual dwarfing genes toward coordinated regulation involving hormone metabolism, cell-wall modification, and signal transduction, with gene editing and genomic selection providing tools for combining favorable alleles. Fourth, stem lodging is mechanically governed by the balance between the wind-induced bending moment (*M*_wind_, N·m) and the breaking or resisting moment of the stem (*M_b_*, N·m). This dimensionally consistent framework links plant architecture, stem geometry, and tissue mechanical properties to structural failure, providing a physical basis for quantitative assessment of lodging resistance.

Phenotyping and detection have progressed from manual field measurements toward intelligent sensing. Although manual mechanical tests and visual scoring are limited by low throughput and subjectivity, ground-based mechanical measurements remain essential reference data for calibrating and validating indirect remote-sensing models. UAV platforms equipped with multispectral, hyperspectral, and other sensors have expanded lodging monitoring from point measurements to spatially continuous coverage. Lodging-induced increases in red reflectance and reductions in near-infrared scattering provide a physical basis for combining multiple vegetation indices rather than relying on a single feature. Moreover, the predominantly structural deformation associated with abiotic lodging and the biochemical deterioration that can accompany biotic lodging provide a mechanistic basis for spectral differentiation, pointing toward a transition from lodging-state detection to cause-oriented diagnosis, although robust attribution under mixed field stresses remains unresolved.

Intelligent recognition methods have likewise progressed from manually calibrated thresholds to deep semantic segmentation. Conventional machine-learning algorithms remain valuable in small-to-medium datasets and in applications that require greater interpretability. CNN-based semantic-segmentation architectures, meanwhile, enable precise pixel-level delineation of lodging patches under complex field backgrounds. To address the high annotation burden and limited cross-environment generalization of deep learning, multi-task learning can jointly optimize related objectives such as lodging detection and yield-loss estimation; rule-constrained learning can incorporate agronomic and spatial priors to improve interpretability and physical consistency; and edge computing can support low-latency inference for real-time deployment. Together, these advances are moving the field from isolated lodging recognition toward a cross-scale framework that connects biomechanical mechanisms, observable digital phenotypes, and field-level decision-making. Reliable transfer across genotypes, environments, sensing platforms, and operational scenarios, however, remains a major challenge.

A concise cross-species comparison of major lodging characteristics, phenotyping approaches, sensing technologies, and management strategies in rice and wheat is summarized in **Table 3**.

**Table 3:** Concise comparison of lodging characteristics, phenotyping, sensing, and management in rice and wheat.

Dimension	Rice	Wheat	Representative references
Major lodging modes	Root lodging; stem bending; stem breaking	Mainly stem bending/breaking; root lodging also occurs	Mullangie et al., 2024; Li et al., 2026a; Piñera-Chavez et al., 2016
Key genetic factors	*sd1*, *OsTCP4–SD1*, *SBI*, *DBC3*	*Rht* alleles, *TaERF-A1*	Ruan et al., 2026; Liu et al., 2018; Dai et al., 2026; Van De Velde et al., 2021; Li et al., 2025b
Root/stem mechanical traits	Root plate spread; anchorage force (F_anchor); anchorage resisting moment (M_anchor); culm diameter; wall thickness; section modulus; breaking resistance	Anchorage force (F_anchor); anchorage resisting moment (M_anchor); center-of-gravity height; culm geometry; section modulus; breaking resistance	Piñera-Chavez et al., 2016; Zhang et al., 2021; Zhang et al., 2025; Ren et al., 2026a
Typical phenotyping	Mechanical tests, micro-CT, UAV MS/HS, LiDAR	Mechanical tests, UAV MS/HS, CHM/LiDAR, satellite sensing	Wu et al., 2021; Kumar et al., 2024; Zhou et al., 2026a; Zhang et al., 2026a
Environmental sensitivity	Soil moisture, N input, shading, ozone/warming, typhoon	Planting density, lower-canopy light, N management, wind/rain	Hu et al., 2023; Zhang et al., 2016; Shao et al., 2024; Mizuta et al., 2023
Sensing technologies	UAV RGB/MS/HS, SAR, depth camera, LiDAR, onboard vision	UAV RGB/MS/HS, SAR, LiDAR, satellite and machine vision	Wang et al., 2020; Chauhan et al., 2020; Yao et al., 2025; Zhang et al., 2026b
Management/harvesting maturity	Agronomic prevention relatively mature; intelligent harvesting under active development	Agronomic optimization mature; lodging-aware harvesting/navigation still emerging	Gu et al., 2025; Wang et al., 2026b; Xie et al., 2023; Niu et al., 2025

CHM, canopy height model; DBC3, DWARF AND BRITTLE CULM 3; HS, hyperspectral; LiDAR, light detection and ranging; micro-CT, X-ray micro-computed tomography; MS, multispectral; N, nitrogen; RGB, red, green, and blue; Rht, Reduced height; SAR, synthetic aperture radar; SBI, SHORTENED BASAL INTERNODES; sd1/SD1, semidwarf 1 (OsGA20ox2); TaERF-A1, *Triticum aestivum* ETHYLENE RESPONSE FACTOR A1; UAV, unmanned aerial vehicle.

### 8.2 Current challenges and future directions

Despite substantial progress, lodging research in rice and wheat still faces several interconnected challenges. Root and stem failure are often analyzed separately despite their mechanical coupling at the whole-plant level; the relationships linking cell-wall composition and tissue anatomy to field-scale failure remain difficult to quantify across scales; and heterogeneous measurement protocols, sensor configurations, and evaluation metrics continue to limit cross-study comparability. Although rice and wheat share common biomechanical principles and sensing approaches, systematic cross-species validation also remains limited. At the application level, a further gap persists between lodging detection and actionable decisions, particularly under spatially heterogeneous field conditions. Together, these limitations restrict the transfer of mechanistic knowledge and predictive models across genotypes, environments, sensing platforms, and operational scenarios.

The priorities outlined below constitute a forward-looking research agenda rather than a summary of established practice; several of the proposed approaches remain at the conceptual stage and require experimental validation before field application.

In the near term, priority should be given to measurement standardization, shared data infrastructure, and rigorous validation. Lodging severity requires harmonized grading criteria, while biomechanical measurements should follow unified protocols specifying growth stage, moisture status, loading mode, force-application position, lever arm, and physical units. Open benchmark datasets should span multiple years, locations, cultivars, and environmental conditions and should link multimodal observations, including RGB, multispectral, hyperspectral, LiDAR, and SAR data, to mechanical and biological ground truth. Standardized metadata, dataset partitions, and evaluation metrics are also needed. Model assessment should extend beyond random within-dataset splits to independent cross-site, cross-year, cross-cultivar, and cross-location tests, accompanied by uncertainty estimates that quantify prediction reliability under domain shift. Future lodging phenotyping should therefore move beyond isolated visual or spectral scores toward coordinated measurement of microstructural, organ-mechanical, whole-plant, and canopy-scale traits, with cross-scale phenotypes calibrated against mechanically defined failure states.

Over the medium to long term, research should focus on mechanistically informed cross-scale modeling and closed-loop intelligent management. The approaches discussed in this paragraph, including physics-informed neural networks, digital twins, and agricultural foundation models, remain largely conceptual and are presented as future possibilities rather than as proven technologies. Integrating multi-omics data with anatomical, biochemical, and biomechanical traits could clarify how molecular regulation propagates to whole-plant mechanical failure. Physics-informed neural networks and digital-twin approaches offer promising routes for embedding mechanically defined variables—such as breaking/resisting moment, section modulus, flexural rigidity, and wind-induced bending moment—into predictive models. Agricultural foundation models may further improve transfer across crops and environments when trained on sufficiently diverse multimodal datasets. At the engineering level, multimodal sensing, edge AI, adaptive machinery control, and autonomous navigation should be integrated into perception–decision–execution systems that convert lodging severity, canopy height, and stem orientation into real-time harvesting actions.

Progress toward field deployment should ultimately be evaluated using quantitative and reproducible benchmarks rather than detection accuracy alone. For predictive models, key criteria should include performance under independent external validation, predictive uncertainty, and degradation under domain shift. For intelligent harvesting systems, recommended benchmarks include end-to-end sensing-to-actuation latency, navigation lateral error, header-height tracking error, harvesting loss rate, and field efficiency. Robustness should also be quantified under controlled disturbances representative of real harvesting environments, including dust, vibration, changing illumination, rainfall, canopy occlusion, and intermittent positioning signals. A common evaluation framework spanning mechanical consistency, cross-domain generalization, and field-level operational performance would provide measurable criteria for comparing methods and assessing readiness for practical deployment.

As a problem spanning plant biology, biomechanics, sensing, and agricultural engineering, lodging in rice and wheat is best understood as a connected cross-scale process rather than a collection of isolated disciplinary problems. Genetic and biochemical regulation shapes tissue anatomy and material properties; these traits, together with organ geometry, determine mechanical resistance and whole-plant failure; mechanical failure generates structural and spectral phenotypes at canopy and field scales; and these phenotypes provide the information needed for detection, decision-making, and harvesting action. Future progress will therefore depend on preserving these cross-scale links across measurement, modeling, validation, and engineering deployment. Shared biomechanical protocols, interoperable multimodal datasets, externally validated models, and robust perception–decision–execution systems will be essential for translating advances in lodging biology and phenotyping into reliable tools for lodging-resistant breeding, field monitoring, and autonomous low-loss harvesting.
